# A Mixed Valence Co^II^Co^III^_2_ Field-Supported Single Molecule Magnet: Solvent-Dependent Structural Variation

**DOI:** 10.3390/molecules26041060

**Published:** 2021-02-18

**Authors:** Susanta Hazra, Cyril Rajnák, Ján Titiš, M. Fátima C. Guedes da Silva, Roman Boča, Armando J. L. Pombeiro

**Affiliations:** 1 Centro de Química Estrutural, Instituto Superior Técnico, Universidade de Lisboa, Av. Rovisco Pais, 1049-001 Lisbon, Portugal; fatima.guedes@tecnico.ulisboa.pt; 2 Department of Chemistry, Faculty of Natural Sciences, University of SS Cyril and Methodius, 91701 Trnava, Slovakia; cyril.rajnak@ucm.sk (C.R.); jan.titis@ucm.sk (J.T.); roman.boca@ucm.sk (R.B.); 3 Peoples’ Friendship University of Russia (RUDN University), 6 Miklukho-Maklaya Street, 117198 Moscow, Russia

**Keywords:** Schiff base, mixed valence, organometallic, pH shift, structural variation, single molecule magnet

## Abstract

One-pot reaction of the Schiff base *N*,*N*’-ethylene bis(salicylaldimine) (H_2_L), CoCl_2_.6H_2_O, and [Ph_2_SnCl_2_] in acetone produces the mixed valence Co^II^Co^III^_2_ compound [Co^II^Co^III^_2_(μ-L)_2_(Ph)_2_(μ-Cl)_2_]·(CH_3_)_2_CO·H_2_O (**1**). Our recent study already revealed that the same reaction mixtures in methanol or ethanol produced a heterometallic Sn^IV^Co^III^ (**2**) or monometallic Co^III^ complex (**3**), respectively. Comparison of these organometallic systems shows that the 2,1-intermetallic Ph shift occurs in any of those solvents, but their relevant structural features (mononuclear, dinuclear-heterometallic, and trinuclear mixed valence) are solvent dependent. Geometrical structural rotation is also discussed among the related organometallic Co^II^Co^III^_2_ systems. The AC magnetic susceptibility measurements indicate that **1** is a single molecule magnet (SMM), exhibiting a field-induced slow magnetic relaxation with two modes. The relaxation time for the low-frequency channel is as slow as *τ*~0.6 s at *T* = 2.0 K and *B*_DC_ = 1.0 T.

## 1. Introduction

The molecules of single molecule magnets (SMMs) [1,2,3,4,5] exhibit slow relaxation of magnetization after the removal of the applied magnetic field, and their magnetic memory originates exclusively from the molecule itself, in contrast to the conventional bulk magnets and molecule-based magnets. The area of SMMs has been a frontier research field because of their potential application in high-density data storage devices, quantum computing, spintronics devices, and magnetic refrigeration [6,7,8,9,10,11,12,13,14,15,16].

Since the discovery of the first SMM, a “Mn_12_” cluster [17,18,19,20], the studies on cluster-based SMMs of 3d metal ions have been a matter of great attention. In fact, several manganese (Mn^III^/Mn^II^/Mn^IV^) systems with Mn^III^ as the common counterpart emerged as SMMs, as well as compounds with other 3d-metal ions, e.g., Cu^II^, Ni^II^, Co^II^, Fe^III^, etc., [21,22,23,24,25,26,27,28,29,30,31]. Both experimental and theoretical investigations have been carried out on several systems and resulted in significant enhancements of SMM-operating temperatures to above 60 K with a high *U*_eff_ [32,33,34,35]. Further explorations of such systems also required to understand their complicated magnetic relaxation mechanism. To be noted that SMMs with more than one relaxation modes have recently been prioritized for investigation [36,37,38,39,40,41]. Particularly, the family of cobalt(II) complexes has been investigated for SMM properties because of the high magnetic anisotropy and strong spin-orbit coupling of cobalt(II) ion [42,43,44,45,46,47,48,49,50,51].

**Scheme 1:**
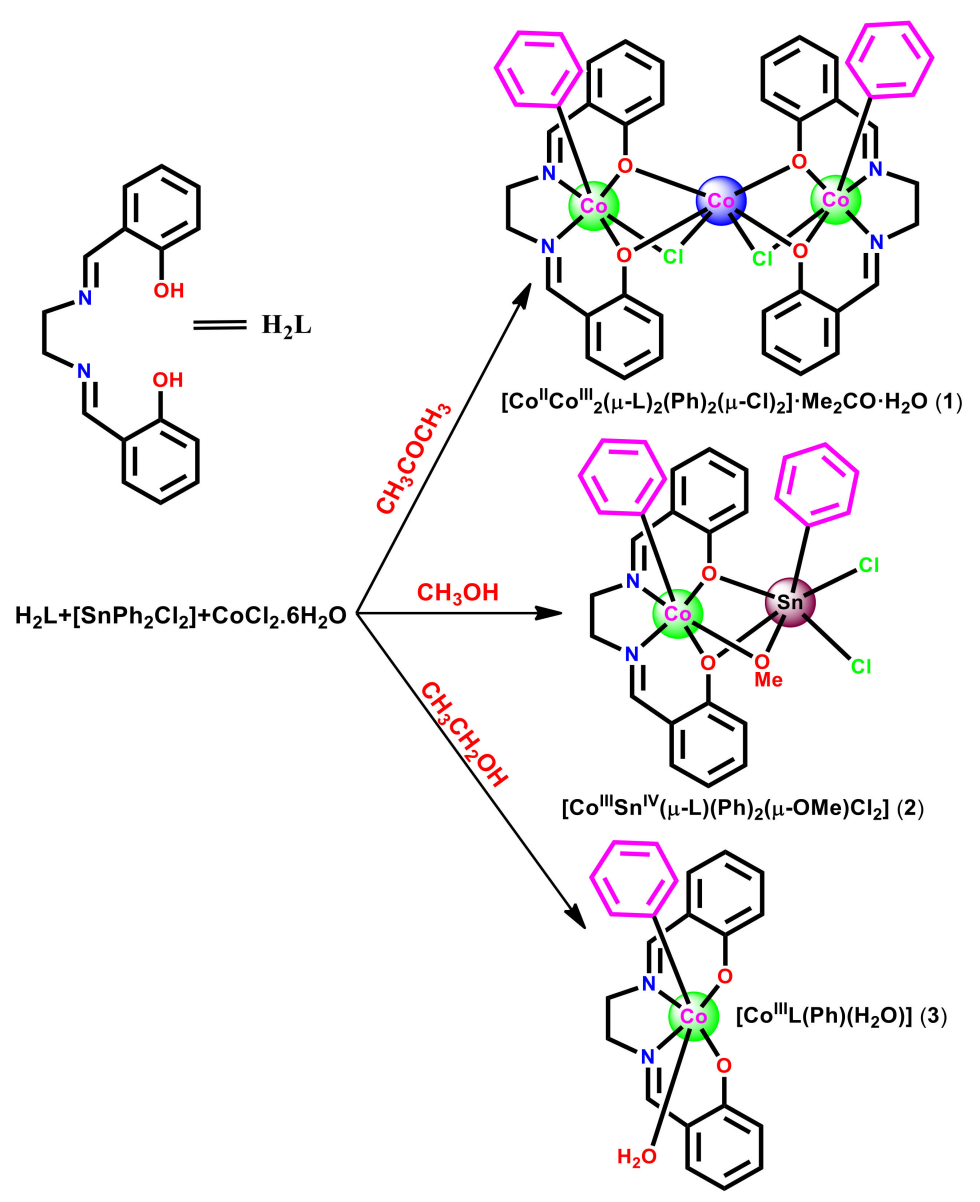
Solvent dependent synthesis of **1**, **2**, and **3** [55].

We have started a structural exploration of heterometallic 3d metal–Sn(II/IV) compounds derived from compartmental Schiff bases under aerobic conditions [3d metal = Co/Ni/Cu] [52,53,54,55]. Our recent study [55] revealed that the open atmosphere self-assembly reaction of *N*,*N*′-ethylenebis(salicylaldimine) (H_2_L) with CoCl_2_·6H_2_O and [SnPh_2_Cl_2_] in MeOH or EtOH produces the hetero-organometallic Co^III^Sn^IV^ system [Co^III^Sn^IV^(μ-L)(Ph)_2_(μ-OMe)(Cl)_2_] (**2**) or the mononuclear organocobalt(III) compound [Co^III^L(Ph)(H_2_O)] (**3**), respectively, generated by a 2,1-intermetallic phenyl shift (Scheme 1) [55]. Continuing our investigation to understand the role of solvents and obtain further types of compounds formed upon such a Ph shift from the organotin reagent, we attempted the reaction in a similar polar solvent (acetone) but with a lower bridging capacity. Interestingly, we have obtained the mixed valence organometallic system [Co^II^Co^III^_2_(μ-L)_2_(Ph)_2_(μ-Cl)_2_]·(CH_3_)_2_CO·H_2_O (**1**) (Scheme 1). Herein, we report its synthesis, crystal structure, and single molecule magnetic properties. In addition, structural comparisons with relevant (**2** and **3**) and related (Co^II^Co^III^_2_) compounds are also discussed.

## 2. Results and Discussion

### 2.1. Synthesis and Characterization

The single compartment Schiff base *N*,*N*’-ethylenebis(salicylaldimine) (H_2_L) (Scheme 1) was synthesized following the reported procedures [56] and, before use, its characterization was confirmed by NMR and elemental analysis [56]. We have already reported that the self-assembly reaction of H_2_L with CoCl_2_·6H_2_O and [SnPh_2_Cl_2_] in alcoholic solvents produced either the hetero-organometallic Co^III^Sn^IV^ system [Co^III^Sn^IV^(μ-L)(Ph)_2_(μ-OMe)(Cl)_2_] (**2**) (in MeOH) or the mononuclear organocobalt(III) compound [Co^III^L(Ph)(H_2_O)] (**3**) (in EtOH), generated upon 2,1-intermetallic Sn-to-Co shift of a phenyl group (Scheme 1) [55]. In the current study, the reaction in boiling acetone under open air atmosphere produces the mixed valence organometallic system [Co^II^Co^III^(μ-L)_2_(Ph)_2_(μ-Cl)_2_]·(CH_3_)_2_CO·H_2_O (**1**) (Scheme 1). Compound **1** was isolated in good yield (62%) from the reaction of the Schiff base and the metal compounds (H_2_L, CoCl_2_·6H_2_O, and [SnPh_2_Cl_2_]) in the 1:1:1 ratio, but a better yield (78%) was obtained with a lower Schiff base amount (1:2:2). Under open air and solvent boiling conditions, oxidation of Co^II^ into Co^III^, followed by the 2,1-intermetallic phenyl shift from tin(IV) to cobalt(III) would result in a {Co^III^(Ph)Sn^IV^(Ph)} moiety as observed in the case of **2** [55]. Further replacement of the organotin(IV) moiety by CoCl_2_ would generate the mixed valence system **1**. Analogous reactions with NiCl_2_·2H_2_O or CuCl_2_·6H_2_O, instead of CoCl_2_·6H_2_O, were attempted but were not successful. Even the reaction of **2** or **3** with CoCl_2_·6H_2_O in refluxing acetone did not produce **1**.

In the IR spectrum, the title compound **1** exhibits a strong intense absorption at 1645 cm^–1^ because of ν(C=N). Medium intense bands at 444 and 459 cm^–1^ can be assigned to ν(Co-N) and ν(Co–O), respectively. An additional broad peak at 3480 cm^–1^ is observed for the non-coordinated water molecule. It was also characterized by elemental analysis and single crystal X-ray diffraction.

### 2.2. Description of Crystal Structure

**Figure 1:**
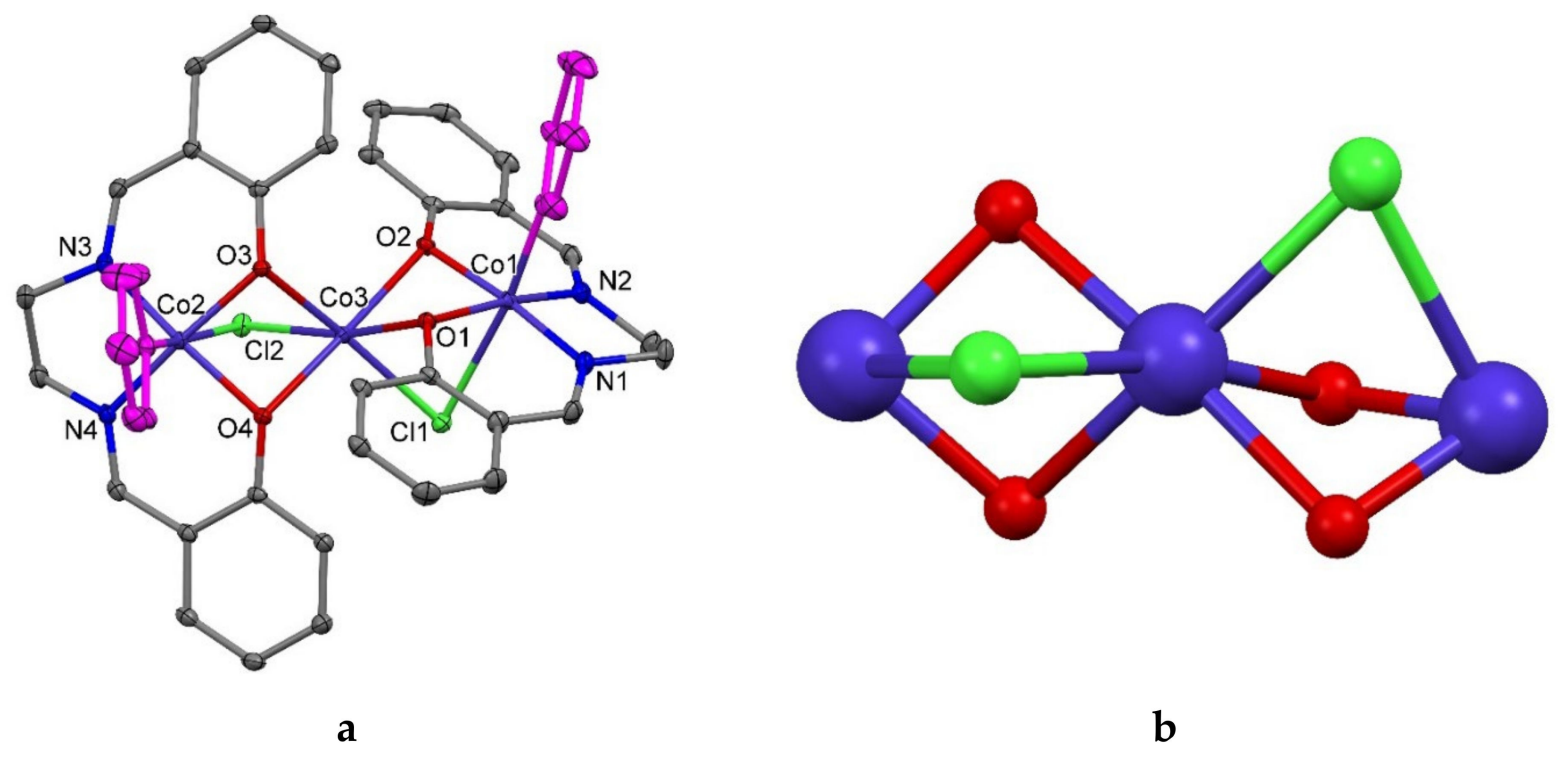
(**a**) Ellipsoid plot (with 30% probability, data collected at 150 K) of the crystal structure of [Co^II^Co^III^_2_(μ-L)_2_(Ph)_2_(μ-Cl)_2_]·(CH_3_)_2_CO·H_2_O (**1**) with partial atom labelling scheme. Solvent molecules and H-atoms are omitted for clarity. (**b**) Representation of the Co^II^-corner shared Co_2_O_2_Cl trigonal bipyramids.

The crystal structure of the mixed valence complex [Co^II^Co^III^_2_(μ-L)_2_(Ph)_2_(μ-Cl)_2_]·(CH_3_)_2_CO·H_2_O (**1**) was determined at 298 and 150 K (see below) and is presented in Figure 1, while some important geometrical parameters are listed in Table 1. As shown in Figure 1, two Co^III^ ions occupy the N_2_O_2_ cavities of the tetradentate Schiff base ligand (L^2−^) while the Co^II^ ion interconnects them through bis(μ-phenoxido) and μ-chlorido bridges. While the peripheral Co(III) metal cations possess CClN_2_O_2_ octahedral geometries with angle variances of ca. 30º^2^, that of the central Co(II) is constructed from two Cl and four O-atoms and is considerably more distorted in view of the measured angle variances of ca. 144–147º^2^. The octahedral volumes of 10.5–10.7 Å^3^ against 13.6 Å^3^ confirm the oxidation states (+3 and +2, in this order) of the metals. Bridging atoms (O and Cl) along with the metal centers (Co^II^ and Co^III^) form two corner-shared (the Co^II^ central cation) Co_2_O_2_Cl trigonal bipyramids (Figure 1b). The Co^III^ cations are displaced by 0.11–0.12 Å from the corresponding least-square N_2_O_2_ planes and toward the phenyl ligands. Such least-squares planes of the L^2−^ ligands in **1** make an angle of 74.14°.

As expected from the metal radii (*r*_Co_^3+^ < *r_Co_^2+^*), the M-O distances involving the Co^II^ center are longer [2.092(2)–2.125(2) Å] than those involving Co^III^ [1.894(2)–1.904(2) Å]; the Co^III^–N lie in the range of 1.862(2)–1.871(2) (Table 1). In contrast, the Co^II^–Cl bond distances are shorter than the Co^III^-Cl (Table 1). The Co–O–Co angles (89.32(6)–90.51(6)°) are larger than the Co–Cl–Co ones (68.71(3) and 69.63(3)°). All the *trans* angles around the metal centers in **1** are lower than 180°.

Most of the geometrical parameters (bond lengths and bond angles) involving the metal centers are typical (Table 1) and observed in other similar mixed valence Co^II^Co^III^_2_ compounds found in the Crystallographic Structural Database [57,58,59].

### 2.3. Solvent Dependent Structural Variation and Rotation of Coordination Geometry

Structural variations that depend on solvent are not uncommon but always interesting [55]. Distinct solvents have different properties, e.g., in terms of polarity, acidity or basicity, coordinating or bridging ability, etc. Cases are also known where solvents do not affect the basic structure, forming solvatomorphs [60,61].

**Figure 2:**
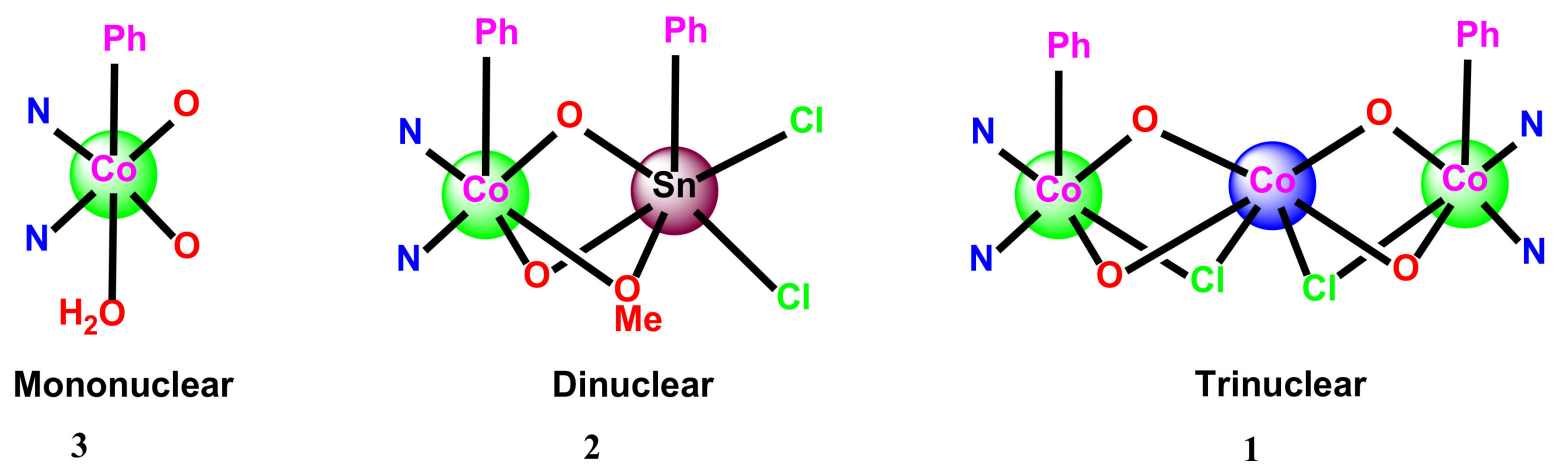
Differences in nuclearity of the organometallic systems **3**, **2**, and **1**.

Heterometallic 3d-tin systems synthesized under open air atmosphere show a structural diversity which includes: i) adducts, ii) non-adduct covalent compounds, iii) cocrystals, iv) salt cocrystals, v) salts, or even vi) hetero-organometallic compounds [52]. In the present work, we have found that the mixed valence Co^II^Co^III^_2_ compound (**1**) was isolated in acetone from a reactant mixture which was used to synthesize other compounds (**2** or **3**) in different solvents (methanol or ethanol) [55]. The 2,1-intermetallic Ph shift (Sn^IV^ to Co^III^) is a common phenomenon in all the cases (**1**, **2,** and **3**), occurring in all the tested solvents, while the Sn^IV^ center of the methanol product (**2**), which is absent in ethanol (in **3**), is formally replaced by CoCl_2_ in acetone, which bridges to another Ph-Co^III^ site generating **1**. As presented in Figure 2, compound **3** is a mononuclear organocobalt(III) and **2** is a dinuclear organocobalt(III)-organotin(IV) compound, while the title compound (**1**) is a trinuclear mixed valence organocobalt(III)-cobalt(II) system. Therefore, not only the nuclearity changes (1→2→3), but also the metal oxidation state and/or combination (Co^III^→Co^III^Sn^IV^→Co^II^Co^III^_2_) differ from one solvent to another, and such a solvent-dependent behavior, to our knowledge, was not known in tin(II/IV) systems derived from the same reactants mixture. In addition, solvents display herein three different roles: coordinated (water in **3**), bridging (methanol derived methoxido in **2**), and non-coordinated (acetone and water in **1**, not shown in Figure 2).

**Figure 3:**
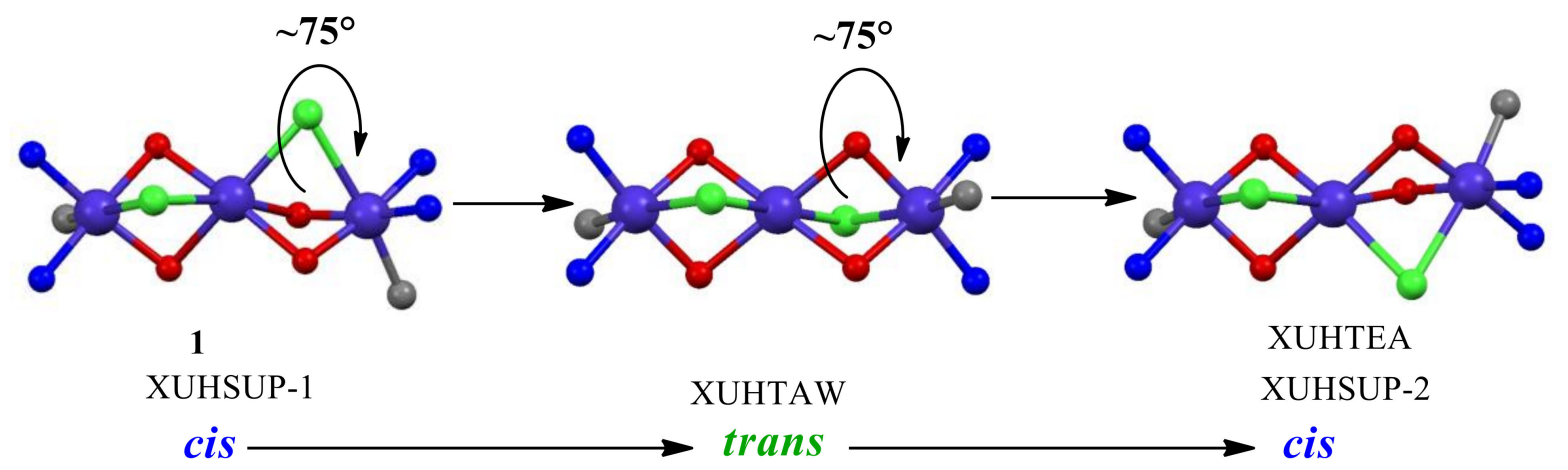
Rotation (by~75°) in geometrical conversion of mixed valence Co^II^Co^III^_2_ systems. Crystal structure of XUHSUP has two isomeric units, presented as XUHSUP-1 and XUHSUP-2 [57,58,59].

A search on the Crystallographic Structural Database [59] shows a considerable number (~300) of compartmental Schiff base cobalt derivatives, ca. 100 of which involve trinuclear species. Further restriction to only dichloro-diphenoxido bridges results in only four systems (CCDC ref. code: AKEHIJ, XUHSUP, XUHTAW, and XUHTEA, Figure S1, Supplementary Materials) [57,58]. AKEHIJ is a Co^II^_3_ system but the remaining three are Co^II^Co^III^_2_ systems containing Co^III^–C bonds (Table 1). In complex **1**, as in XUHSUP and XUHTEA, the chloride anions are in *cis* position while in XUHTAW they are mutually *trans*. Our compound confirms that non-bulky ligands (phenyl groups in **1**), to complete the geometry of the peripheral cobalt cations, favor their *cis* geometry [57,58]. Upon redrawing their metal coordination geometries by keeping one of the outer octahedra fixed, we observe that the other outer octahedron has rotated by ca. 75° in the sequence **1**, XUHSUP-1 (*cis*) to XUHTAW (*trans*) to XUHTEA, XUHSUP-2 (*cis*) (Figure 3). Such a structural relation in mixed valence trinuclear Co^II^Co^III^_2_ systems is worthy to be mentioned [59].

### 2.4. Magnetic Properties

Mixed valence compounds are also very important in the field of magnetism [20,24,27,46,62,63,64,65,66,67,68]. Their magnetic properties are usually investigated as they can show interesting magnetic exchange coupling (ferro or antiferro) or slow magnetic relaxation (characteristic of a single molecule magnet, SMM). The majority of mixed valence SMM systems is based on manganese(IV/III/II) [20,24,64,65,66,67,68]. Although cobalt(II)-containing compounds can also display single molecule magnetism, known as mixed valence cobalt(II/III) SMM systems are only a few [45,46] and one of them shows multiple magnetic relaxation [46]. Therefore, the investigation of the magnetic behavior of such systems is promising and we have now studied both the DC and AC magnetic properties of our trinuclear mixed valence Co^II^Co^III^_2_ system **1**. However, the outer Co(III) ions in **1** are expected to be diamagnetic because of the low spin d^6^ electronic configuration.

#### 2.4.1. DC Magnetic Data

**Figure 4:**
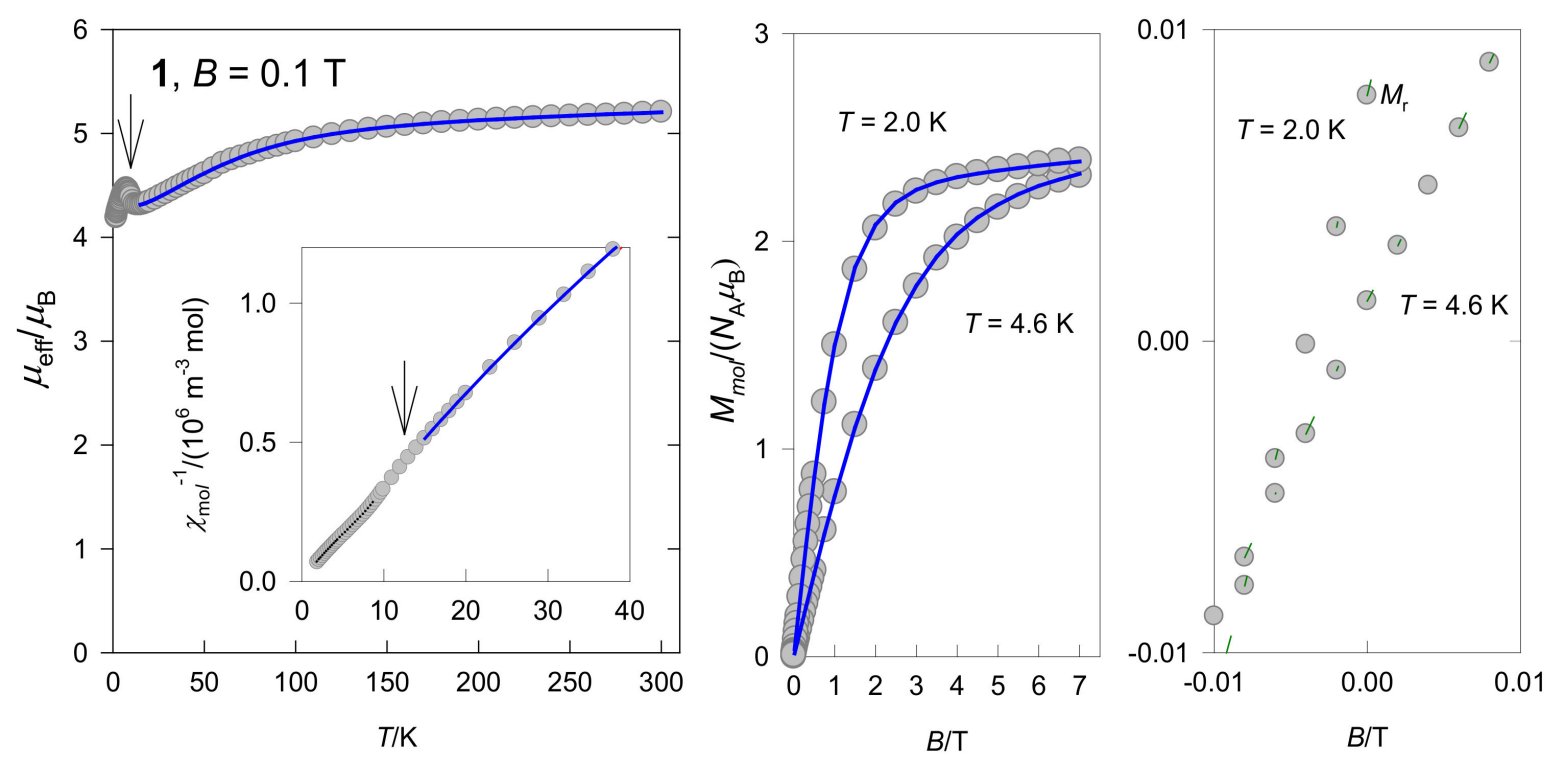
DC magnetic functions for **1**. Inset: inverse molar magnetic susceptibility. Solid lines—fitted susceptibility data above 15 K according to the ZFS model; dashed—linear regression for the data below 8 K. Arrow indicates an anomaly.

The temperature evolution of the effective magnetic moment displays features that are typical for a single hexacoordinate Co(II) complex with large zero-field splitting (Figure 4). The room-temperature value *μ*_eff_ = 5.20 *μ*_B_ on cooling gradually decreases to *T*~15 K and then it shows a hook. The magnetization per formula unit at *B* = 7.0 T and *T* = 2.0 K saturates to *M*_1_ = *M*_mol_/*μ*_B_ = 2.39 that is far from the spin-only value owing to the zero-field splitting. There is a small remnant magnetization *M*_r_ at both, *T* = 4.6 and 2.0 K, confirming a long-range ordering. This causes a hook visible at the low-temperature effective magnetic moment. The inverse susceptibility below 15 K turns toward zero, however on further cooling it returns back to the linear dependence.

Both magnetic data-sets were fitted simultaneously by minimizing a functional of the form $F=w\cdot E\left(\chi \right)+\left(1-w\right)\cdot E\left(M\right)$ that balances the relative errors of the susceptibility and magnetization, respectively. A zero-field splitting (ZFS) Hamiltonian working in the space of spin-only kets

(1) $${\hat{H}}_{a}^{\mathrm{eff}}=D\left({\hat{S}}_{z}^{2}-{\vec{S}}^{2}/3\right)\hbar^{-2}+\mu_{B}B\left(g_{z}{\hat{S}}_{z}\cos\theta_{a}+g_{xy}{\hat{S}}_{x}\sin\theta_{a}\right)\hbar^{-1}$$

has been used with the axial zero-field splitting parameter *D*. The Zeeman term mimics the powder-sample property: it depends upon the distribution of the magnetic field along a set of uniformly separated grids at the meridian. The eigenvalues in the basis set of spin kets enter the formulae of the statistical thermodynamics for the magnetization and susceptibility, respectively [69]. The fitting procedure was restricted to the susceptibility data above 15 K and it gave the following set of magnetic parameters: *D*/*hc* = 86.9 cm^−1^, *g_z_* = 2.030, *g_xy_* = 2.778, the molecular field correction *zj*/*hc* = +0.10 cm^−1^, and the temperature-independent magnetism *χ*_TIM_ = 10 × 10^−9^ m^3^ mol^−1^. The fit is perfect as the discrepancy factors *R*(*χ*) = 0.0011 and *R*(*M*) = 0.0061. The g-factors average to *g*_av_ = 2.53.

A more elaborate Griffith–Figgis model working in the space of spin-orbit kets

(2) $$\begin{array}{cc} {\hat{H}}^{GF} & =-A\kappa \lambda ({\vec{L}}_{p}\times \vec{S})h^{-2}+\mu_{B}\vec{B}\times (g_{e}\vec{S}-A\kappa {\vec{L}}_{p})h^{-1}\\ & +[\Delta_{ax}({\hat{L}}_{p,z}^{2}-{\vec{L}}_{p}^{2}/3)+\Delta_{rh}({\hat{L}}_{p,x}^{2}-{\hat{L}}_{p,y}^{2})]h^{-2} \end{array}$$

involves the spin-orbit splitting parameter *λ* = −*ξ*/2*S*, the orbital reduction factor *κ*, the Figgis CI parameter *A*, and the axial and rhombic splitting parameters Δ_ax_ and Δ_rh_, respectively. Owing to the T-p isomorphism the orbital angular momentum refers to *L*_p_ = 1 [69]. The susceptibility and magnetization data were fitted with a reasonable set of magnetic parameters: (*Aκλ*)/*hc* = −170 cm^–1^, *g*_L_ = −(*Aκ*) = –1.44, Δ_ax_/*hc* = −650 cm^−1^, Δ_rh_/*hc* = 25 cm^–1^, *zj*/*hc* = 0.063 cm^−1^ and *χ*_TIM_ = 11 × 10^−9^ m^3^ mol^−1^; *R*(*χ*) = 0.0017 and *R*(*M*) = 0.032. With negative Δ_ax_ the ground state refers to the E_g_ crystal field term that is further split by Δ_rh_ to a pair of orbitally non-degenerate terms. The results are displayed in SI.

#### 2.4.2. Ab Initio Calculations

The central atom of Co(II) in the present trinuclear assembly possesses a highly distorted geometry. Three pairs of along the *trans* ordinate display distances {Co3-O4 2.124, Co3-O2 2.125}, {Co3-Cl2 2.409, Co3-O1 2.114}, and {Co3-Cl1 2.401, Co3-O3 2.092} in Å. Therefore, it is expected that the energy spectrum will not contain orbitally degenerate terms.

The results of the ab initio calculations using the ORCA package at the CASSCF+NEVPT2 level gave the data as listed in Table 2. The results need to be discussed at three levels of sophistication.

First, the CASSCF calculations gave energies of the multielectron terms. In the light of the crystal field theory, the ground octahedral term ^4^T_1g_ is split on the symmetry lowering to the tetragonally elongated system into the ^4^E_g_ (ground) and ^4^A_2g_ (excited) terms. The orbital degeneracy of the ^4^E_g_ term is lifted on further symmetry lowering. To this end, the calculated energies of the multielectron terms are interpreted according to the following scheme:

(3) 4T1g→→A42g→3A41318 cm−1→E4g→→2A4394 cm−1→1A40,ground

The involvement of the spin-orbit interaction causes the splitting of the multielectron terms into multiplets (Kramers doublets) with the relative energies {0, 195, 645, 952} and {1653, 1763} where the first bracket arises from the mother ^4^E_g_ term and the second one from ^4^A_2g_. It need be emphasized that four lowest Kramers doublets have no relationship to the spin Hamiltonian parameters such as *D* and *E* because they result from the pair of quasidegenerate terms 1^4^A and 2^4^A for which the perturbation theory diverges. The multiplet splitting *δ* = 195 cm^−1^ needs to be considered as a principal and valuable result of the calculations.

#### 2.4.3. AC Susceptibility Data

**Figure 5:**
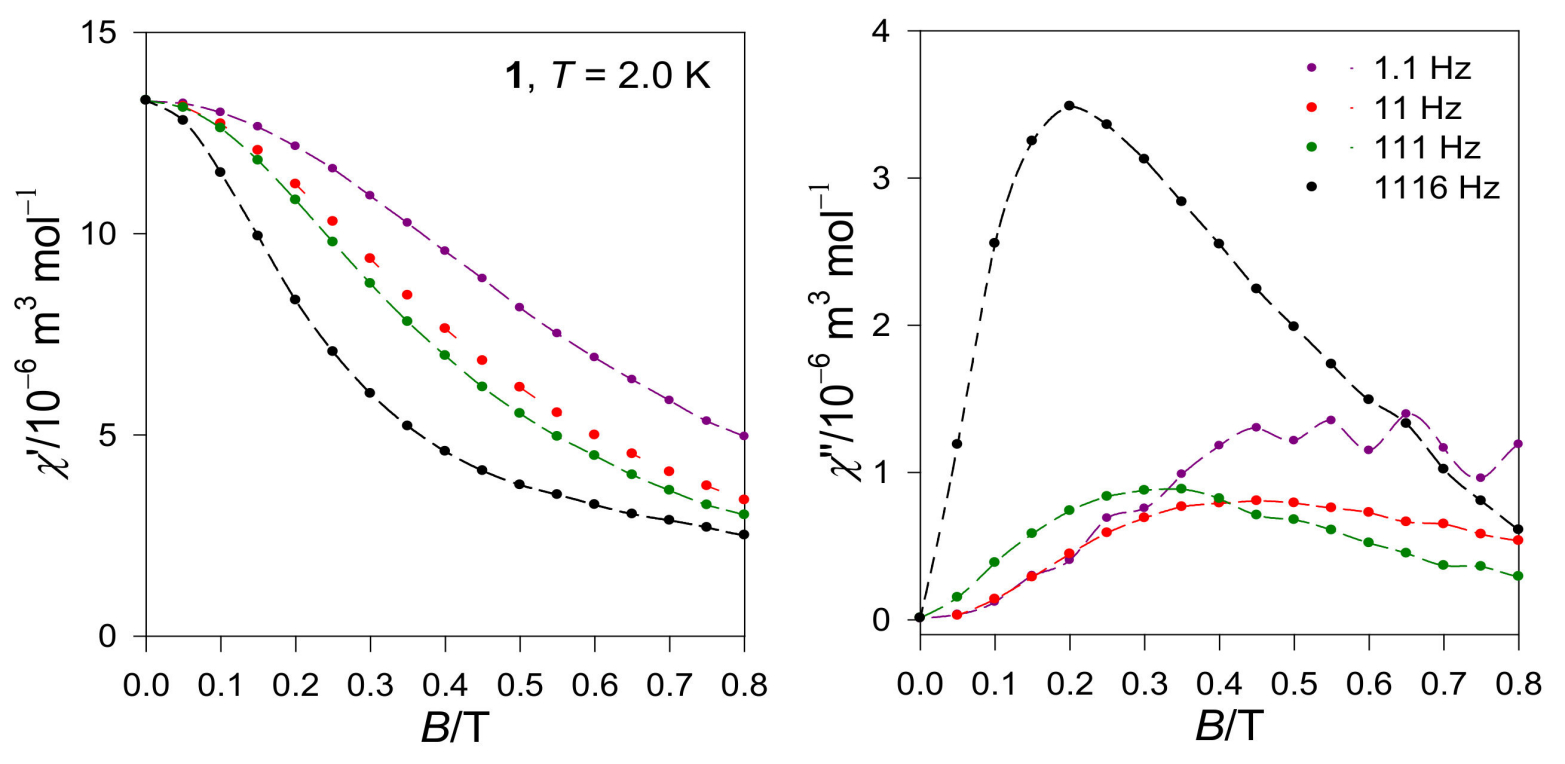
AC susceptibility data for **1**: field dependence for a representative set of frequencies at *T* = 2.0 K. Lines are visual guide.

In the first scan the AC susceptibility response is investigated at low temperature, depending upon the external DC field, for a set of representative frequencies *f* of the AC field (Figure 5). The out-of-phase susceptibility *χ*’’ is silent at the zero field, however, it rises with the applied field, passes through a maximum, and then attenuates. Such a behavior confirms that **1** shows a field-induced slow magnetic relaxation. The frequency dependence of maxima at *χ*’’ indicates an existence of two or more relaxation channels.

**Figure 6:**
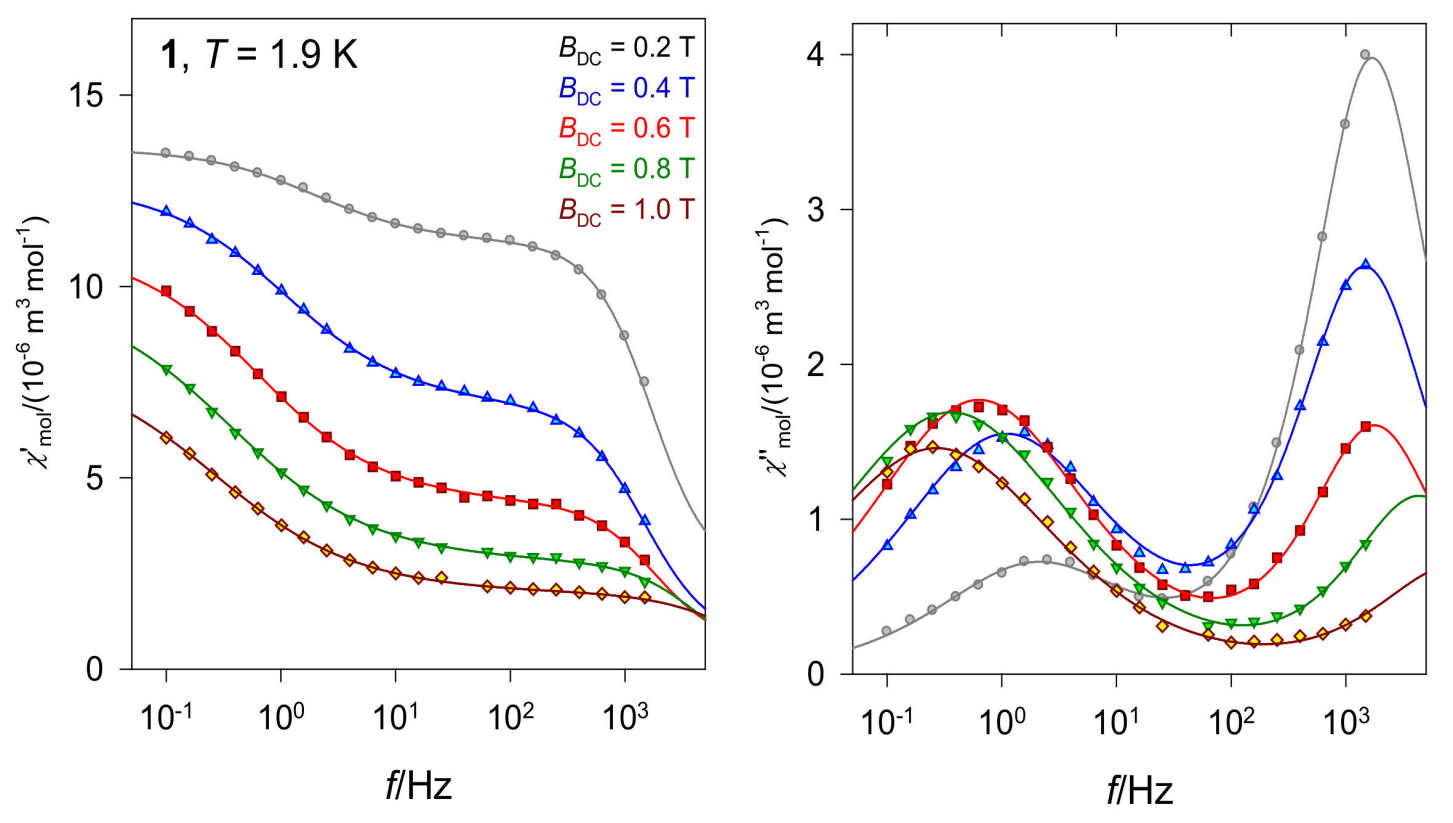
AC susceptibility data for **1**: frequency dependence for a set of the external fields at *T* = 1.9 K. Solid lines—fitted with a two-set Debye model.

The AC susceptibility data, taken for 22 frequencies ranging between *f* = 0.1 to 1500 Hz, are shown in Figure 6 at fixed *T* = 1.9 K and for an external magnetic field up to *B*_DC_ = 1.0 T. Two maxima of the out-of-phase susceptibility confirm two relaxation channels: the low-frequency (LF) and the high-frequency (HF). The DC field supports the LF channel and shifts the relaxation time $\tau =1/(2\pi f_{max}^{\prime \prime }$ to higher values.

The AC susceptibility data (22 points for *χ*′ and 22 points for *χ*″) have been subjected to the fitting procedure by employing the extended two-set Debye model

(4) $$\chi \left(\omega \right)=\chi_{S}+\overset{2}{\underset{k=1}{\sum }}\frac{\chi_{Tk}-\chi_{S}}{1+{\left(i\omega \tau_{k}\right)}^{1-\alpha_{k}}}$$

containing seven free parameters: the isothermal susceptibilities *χ_Tk_*, the distribution parameters *α_k_*, and the relaxation times *τ_k_* for each relaxation channel, along with the adiabatic susceptibility *χ_S_*. This equation allows separating the real (in-phase) and the imaginary (out-of-phase) components. A joint functional $F=w\cdot E\left(\chi^{\prime }\right)+\left(1-w\right)\cdot E\left(\chi^{\prime \prime }\right)$ is minimized during the fitting process. The final relaxation parameters with their standard deviations, and the discrepancy factors of the fit, are presented in Table 3. These parameters were used in creating extrapolation/interpolation lines shown in Figure 6. Notice, at *B*_DC_ = 1.0 T and *T* = 1.9 K the relaxation time is as long as *τ*_LF_ = 0.59(3) s and the mole fraction is *χ*_LF_ = 0.76.

Temperature evolution of the AC susceptibility components at fixed *B*_DC_ = 0.6 T is shown in Figure S2 (Supplementary Materials) for a set of frequencies *f* = 0.1–1500 Hz. The individual frequency components *χ*’ merge at *T* > 5 K that can be assigned as a blocking temperature; simultaneously *χ*’’ vanishes.

**Figure 7:**
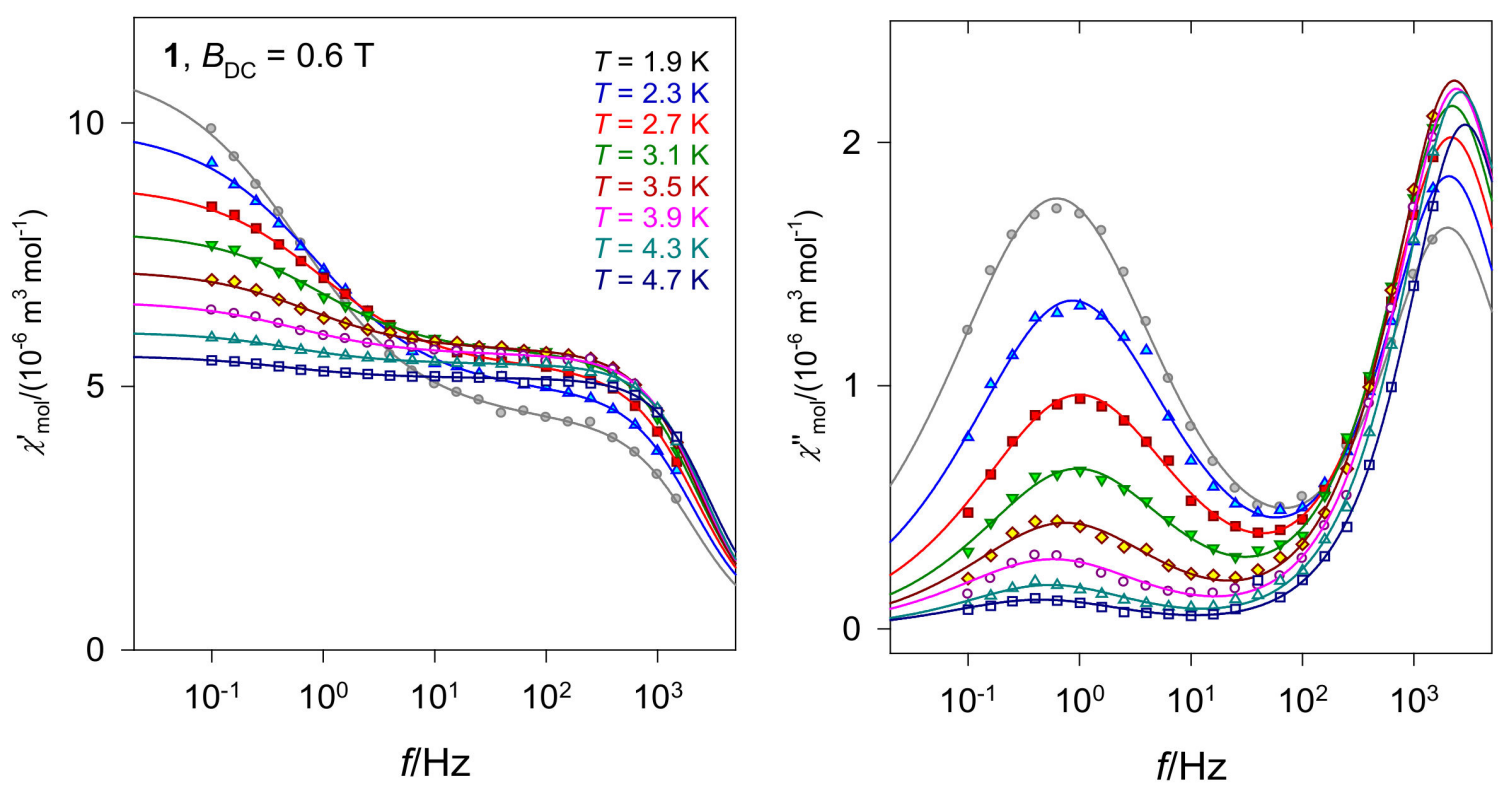
Frequency dependence of the AC susceptibility components of **1** for a set of temperatures at *B*_DC_ = 0.6 T. Solid lines—fitted with a two-set Debye model.

The subsequent data acquisition was done at the external field *B*_DC_ = 0.6 T for a set of temperatures (Figure 7). Based upon the profile of the *χ*’’ *vs f* [*T*, *B*_DC_] curves, two relaxation channels are well recognized. On heating, the LF peak progressively decays which is confirmed by the fitted values of *χ*_LF_ and *x*_LF_ (Table 4). Above *T* > 4.7 K the LF-*χ*’’ component becomes too flat, and the HF tail spans outside the limit of the data taking; these features prevent a reliable data fitting.

**Figure 8:**
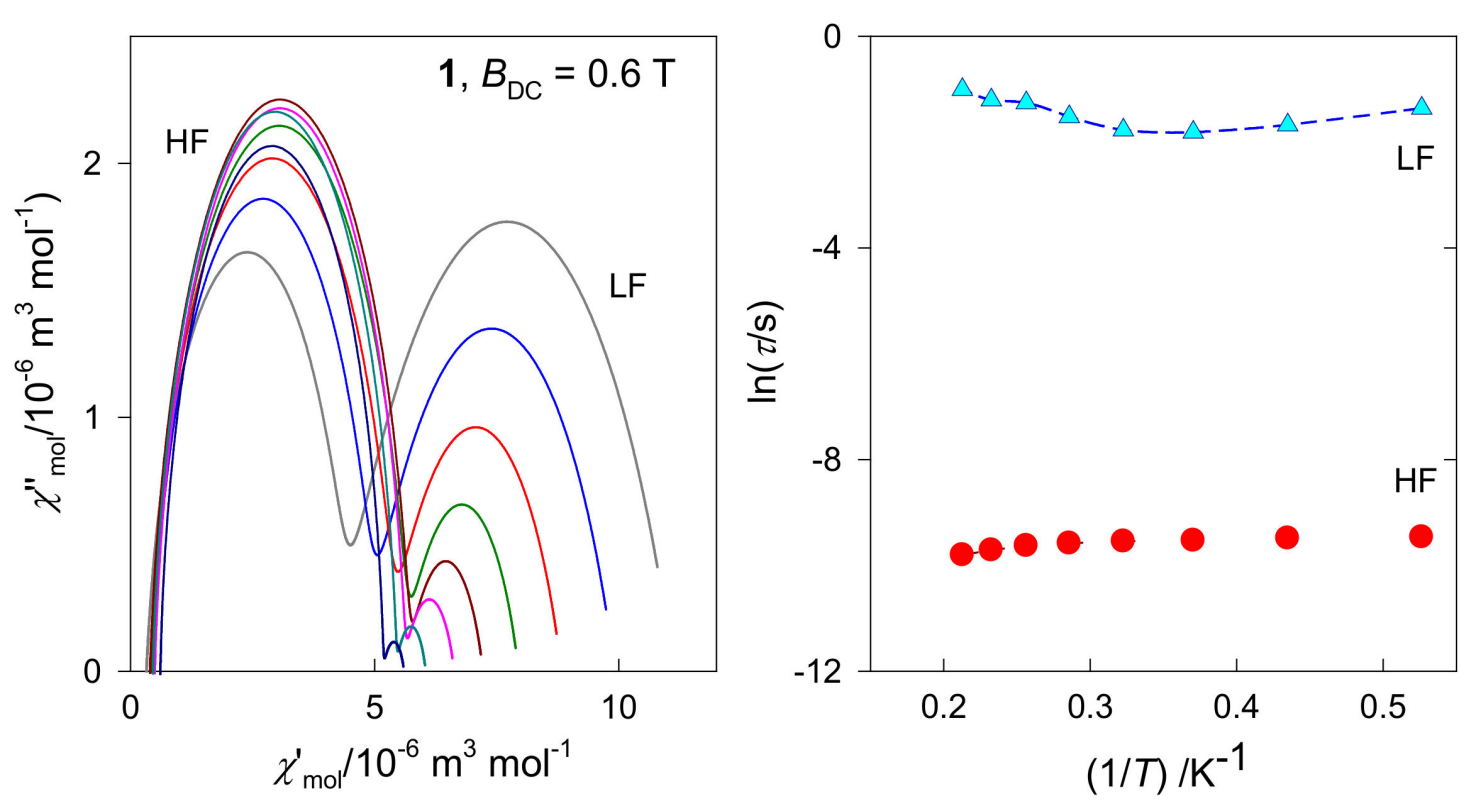
Fitted AC susceptibility data for **1**: Argand diagram (**left**) and Arrhenius-like plot (**right**). Lines are guide for eyes.

The Argand (Cole-Cole) diagram and the Arrhenius-like plot are presented in Figure 8. The Argand diagram is formed of two overlapping arcs of different height, position, and flattening. The Arrhenius-like plot ln*τ vs T*^−1^ maps a temperature evolution of the LF and HF relaxation times. The relaxation time referring to the high-frequency relaxation channel decreases with temperature only slightly.

**Figure 9:**
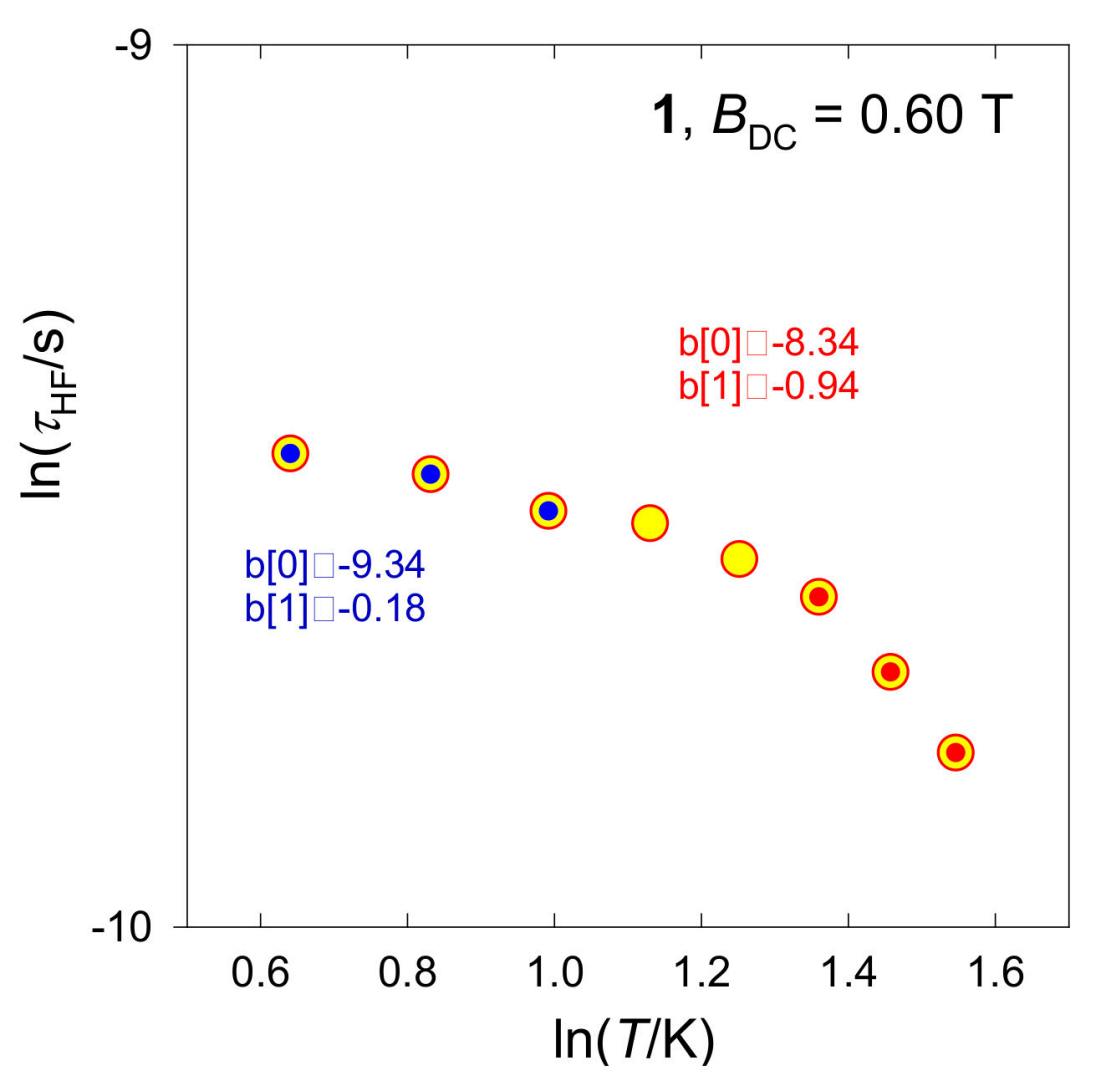
Thermal development of the relaxation time for the high-frequency relaxing species of **1**.

In order to analyze the relaxation time for the high-frequency relaxing species of **1**, Figure 9 was constructed. Three high-temperature and three low-temperature points were fitted via linear regression. They refer to an individual relaxation equation $\tau^{-1}=CT^{n}$ which yields the linearized form $ln\tau =-lnC-n\cdot lnT=b\left[0]+b[1\right]\cdot lnT$. The high-temperature points gave *n*~1 which is a fingerprint of the direct process of the relaxation that is usually written as $\tau^{-1}=AB^{m}T=A_{B}T$ (For the Raman process *n* = 7–9 are typical values). The second straight line possess a very small slope so that probably the temperature-independent quantum tunneling process $\tau^{-1}=D\left(B\right)$ adopts its significance.

## 3. Materials and Methods

All the reagents and solvents were purchased from commercial sources and used as received. The Schiff base H_2_L was prepared according to the reported procedures [56]. Elemental (C, H, and N) analyses were performed on a Perkin-Elmer 2400 II analyzer. IR spectra were recorded in the region 400–4000 cm^–1^ on a Perkin-Elmer RXIFT spectrophotometer with samples as KBr disks.

### 3.1. Synthesis

To an acetone suspension (20 mL) of H_2_L (0.134 g, 0.5 mmol) were added CoCl_2_·6H_2_O (0.120 g, 0.5 mmol) and [SnPh_2_Cl_2_] (0.172 g, 0.5 mmol). The resulted reaction mixture was refluxed for 30 min to obtain a dark red solution which was kept at room temperature for slow evaporation. Within 3 h, the formed dark red crystals, suitable for X-ray diffraction analysis, were collected by filtration and washed with cold acetone. Other fractions were then obtained from the reaction mixture, one of those sent for elemental analysis. Total yield: 0.156 g (62%). C_47_H_46_Cl_2_Co_3_N_4_O_6_ (1010.60): calcd. C 55.86, H 4.59, N 5.54%; found. C 55.11, H 4.53, N 5.62%. IR data (KBr, cm^−1^): ν(HO), 3480br; ν(C = N), 1645s; ν(Co–O), 459 m; ν(Co–N), 444 m.

### 3.2. Crystal Structure Determination

An X-ray quality crystal of **1** was immersed in cryo-oil, mounted in a Nylon loop and measured at 150 K and 298 K. Intensity data were collected using a Bruker APEX II SMART CCD diffractometer with graphite monochromated Mo-Kα (*λ* = 0.71073 Å) radiation. Data were collected using omega scans of 0.5º per frame and full sphere of data were obtained. Cell parameters were retrieved using Bruker SMART software and refined using Bruker SAINT [70] on all the observed reflections. Raw data were corrected for absorption effects using the multi-scan method (SADABS) [70]. Structures were solved by direct methods by using the SHELXS-2014 package [71] and refined with SHELXL-2014/7 [71]. Calculations were performed using the WinGX Version 2014.01 [72]. All the hydrogen atoms attached to carbon atoms were inserted at geometrically calculated positions and included in the refinement using the riding-model approximation. *U*_iso_(H) were defined as 1.2 *U*_eq_ of the parent carbon atoms for phenyl and methylene residues and 1.5 *U*_eq_ of the parent carbon atom for the methyl group. Least square refinements with anisotropic thermal motion parameters for all the non-hydrogen atoms were employed. Solvent molecules in the structure of **1** obtained at room temperature were highly disordered and could not be modelled; therefore, they were removed by using *PLATON* SQUEEZE routine [73]. A total void of 1301 Å^3^ containing 312 electrons per unit cell was found and fits well for one acetone (32 electrons) and one water molecule (10 electrons) per asymmetric unit. These results are reasonably consistent with the elemental analysis and the low temperature (150 K) X-ray data collection where such crystallization solvent molecules were located. The final refinement converged to R_1_ (*I* > 2σ(*I*)) value of 0.0334 (at 150 K) and 0.0559 (at 298 K). Crystallographic data of **1** at both 150 and 298 K are summarized in Table S1 (Supplementary Materials).

### 3.3. Magnetic Measurements

The DC magnetic data were taken with a SQUID magnetometer (MPMS-XL7, Quantum Design) using the RSO mode of detection at *B*_DC_ = 0.1 T. Freshly prepared samples were encapsulated in a diamagnetic gelatin-made sample holder. The molar magnetic susceptibility data were corrected for the underlying diamagnetism and presented in the form of the effective magnetic moment. Magnetization data were taken at *T* = 2.0 and 4.6 K until *B*_DC_ = 7.0 T and presented in the form of magnetization per formula unit per Bohr magneton, *M*_1_ = *M*_mol_/(*N*_A_*μ*_B_). The AC susceptibility data were taken at the oscillating field *B*_AC_ = 0.38 mT for 22 frequencies in the range of *f* = 0.1–1500 Hz. In these measurements an external magnetic field *B*_DC_ = 0.2, 0.4, 0.6, 0.8, and 1.0 T was applied at a fixed temperature (*T* = 2.0 K) while at a fixed field (*B*_DC_ = 0.6) AC susceptibility data were for a set of different temperatures (*T* = 1.9–4.7 K).

### 3.4. Theoretical Calculations

Ab initio calculations were performed with ORCA 4.0.0 computational package [74] using the experimental geometry of the complex under study. The relativistic effects were included in the calculations with zero-order regular approximation (ZORA) together with the scalar relativistic contracted version of def2-TZVP basis functions for Co atom and def2-SV(P) basis functions for other elements. The calculations of ZFS parameters were based on state average complete active space self-consistent field (SA-CASSCF) wave functions complemented by N-electron valence second order perturbation theory (NEVPT2) [75,76,77]. The active space of the CASSCF calculations comprised of seven electrons in five metal-based d-orbitals. The state averaged approach was used, in which all 10 quartet and 40 doublet states were equally weighted. The calculations utilized the RI approximation with appropriate decontracted auxiliary basis set and the chain-of-spheres (RIJCOSX) approximation to exact exchange. Increased integration grids (Grid4 and GridX5) and tight SCF convergence criteria were used. The ZFS parameters were calculated through quasi-degenerate perturbation theory in which an approximation to the Breit–Pauli form of the spin-orbit coupling operator (SOMF) and the effective Hamiltonian theory was utilized [78,79,80].

## 4. Conclusions

The mixed valence Co^II^Co^III^_2_ compound [Co^II^Co^III^_2_(μ-L)_2_(Ph)_2_(μ-Cl)_2_]·(CH_3_)_2_CO·H_2_O (**1**) was obtained from the reaction mixture of the Schiff base *N*,*N*’-ethylenebis(salicylaldimine) (H_2_L), CoCl_2_.6H_2_O, and [Ph_2_SnCl_2_] in acetone, while a dinuclear organocobalt(III)-organotin(IV) compound (**2**) and a mononuclear organocobalt(III) (**3**) were isolated from the same reaction mixture but using different alcohols as solvents. The strategy revealed a few interesting points: (i) The occurrence of the 2,1-intermetallic Ph shift (Sn^IV^→Co^III^) is independent of the solvent used; (ii) the solvent-dependent nuclearity of the metal complexes [1 (in **3**)→2 (in **2**)→3 (in **1**)]; (iii) the solvent-dependent combination of metal ions (Co^III^→Co^III^Sn^IV^→Co^II^Co^III^_2_) resulting in homo, hetero, or mixed valence systems. To our knowledge, such a solvent-dependent structural diversity in association with a solvent-independent occurrence of Ph transfer has never been found in tin(II/IV) systems derived from a common reactants mixture.

Further comparison of the title compound **1** with related mixed valence organometallic Co^II^Co^III^_2_ systems shows that their different metal coordination geometries (involving *cis* or *trans* chloro ligands) are related by rotations of one of the outer moieties relatively to the other one.

The AC susceptibility measurements indicate that **1** is a single molecule magnet showing a field-induced slow magnetic relaxation with two relaxation modes. While the high-frequency process spans the usual range [4,5,6,7] of the relaxation time for other single molecule magnets (*τ*_HF_~10^−7^ s), the low-frequency branch is as slow as *τ*_LF_~0.6 s at *T* = 2.0 K and *B* = 1.0 T. While the barrier height and the blocking temperature for **1** are in the range of other reported SMMs [17,18,19,20,21,22,23,24,25,26,27,28,29,30,31,32,33,34,35,36,37,38,39,40,41,42,43,44,45,46,47,48,49,50,51], two relaxation modes were not investigated commonly in a Co^II^Co^III^_2_ mixed valence system.

## Figures and Tables

**Table 1:** Comparison of selected structural parameters (distances in Å and angles in °) of **1** (at 150 and 298 K) and other related Co^II^Co^III^_2_ compounds. Reported compounds are presented by their CCDC reference codes and deposition numbers [59].

	1 (at 150 K)	1 (at 298 K)	XUHSUP	XUHTAW	XUHTEA
	Co^II^Co^III^_2_	Co^II^Co^III^_2_	Co^II^Co^III^_2_	Co^II^Co^III^_2_	Co^II^Co^III^_2_
CCDC	2016605	2016606	717829	717830	717831
Co^III^–C	1.934(2) 1.936(2)	1.930(5) 1.937(4)	1.939(9) 1.948(9) 1.951(1) 2.077(1)	1.965(7)	1.952(7) 2.074(10)
Co^III^–N	1.862(2) 1.864(2) 1.865(2) 1.871(2)	1.850(4) 1.859(4) 1.863(4) 1.868(3)	1.844(8) 1.902(7) 1.879(7) 1.891(8) 1.880(7) 1.895(6) 1.844(8) 1.868(8)	1.879(5) 1.886(4)	1.861(6) 1.864(5) 1.881(5) 1.884(5)
Co^III^–O	1.894(2) 1.896(2) 1.903(2) 1.904(2)	1.894(3) 1.895(3) 1.900(3) 1.906(3)	1.864(5) 1.871(6) 1.872(6) 1.876(5) 1.878(5) 1.889(6) 1.892(6) 1.896(6)	1.875(4) 1.881(3)	1.866(4) 1.879(4) 1.879(4) 1.887(5)
Co^III^–Cl	2.565(6) 2.636(7)	2.573(1) 2.657(1)	2.507(3) 2.532(3) 2.604(3) 2.625(3)	2.561(2)	2.599(2) 2.516(2)
Co^II^–O	2.092(2) 2.114(2) 2.124(2) 2.125(2)	2.092(3) 2.121(3) 2.125(3) 2.138(3)	2.080(6) 2.081(5) 2.083(6) 2.088(6) 2.100(6) 2.136(6) 2.194(6) 2.202(6)	2.091(4) 2.094(4)	2.097(4) 2.104(4) 2.112(4) 2.113(4)
Co^II^–Cl	2.401(7) 2.409(7)	2.395(1) 2.409(1)	2.408(2) 2.409(3) 2.414(3) 2.425(3)	2.441(2)	2.402(2) 2.407(2)
Co^III^···Co^II^	2.835(1) 2.849(1)	2.848(1) 2.859(1)	2.839(2) 2.843(2) 2.850(2) 2.854(2)	2.832(1)	2.835(1) 2.836(1)
N–Co^III^–O (*trans*)	171.25(8) 171.86(8) 172.94(8) 174.67(8)	171.16(1) 171.99(1) 172.80(1) 174.62(1)	171.3(3) 172.3(3) 172.3(3) 172.5(3) 172.9(3) 174.6(3) 174.8(3) 176.7(3)	172.77(19) 172.77(18)	171.5(2) 174.2(2) 175.0(2) 172.3(2)
O–Co^II^–O (*trans*)	172.23(6)	171.95(1)	174.9(2) 177.2(2)	180.0 180.0	176.3(2)
O–Co^II^–Cl (*trans*)	170.20(5) 169.57(5)	169.93(8) 170.79(9)	164.48(16) 164.86(18) 166.95(18) 167.43(17)	-	167.10(13) 167.98(12)
Cl–Co^II^–Cl	105.27(2)	104.80(4)	105.35(9) 109.28(9)	180.0	105.79(6)
Co^III^–O–Co^II^	89.32(6) 90.09(6) 90.17(6) 90.51(6)	89.74(1) 90.12(1) 90.46(1) 91.05(1)	88.2(2) 91.3(2) 90.2(2) 91.7(2) 88.4(2) 92.2(2) 91.2(2) 91.0(2)	90.79(14) 90.87(15)	90.33(16) 90.34(16) 90.58(17) 90.94(16)
Co^III^–Cl–Co^II^	68.712(18) 69.425(18)	68.71(3) 69.63(3)	68.43(7) 70.21(7) 69.17(7) 70.70(7)	68.91(5)	68.86(5) 70.38(5)
Co^III^···Co^II^···Co^III^	158.68	157.94	163.50(6) 163.70(6)	180.0	163.51(4)

**Table 2:** Calculated (CASSCF&NEVPT2) energies and magnetic parameters for complex **1**. ^a^

1. Term energies: quartet-to-quartet transition energies/cm^−1^	{0, 394, 1318}(^4^T_1g_), {7061, 7365, 7630}(^4^T_2g_), 14988(^4^A_2g_), {20620, 20951, 21786}(^4^T_1g_)
2. Effect of SOI: energies of spin-orbit multiplets (Kramers doublets) of the ground term/cm^−1^	0, 195, 645, 952, 1653, 1763
3. Spin Hamiltonian parameters	
*g*_1,2,3_; *g*_av_	1.904, 2.303, 3.012; 2.41
*D*/*hc*/cm^−1^	−85.27
*E*/*D*	0.32

^a^ Assignment of the terms in O_h_ symmetry.

**Table 3:** Field dependence of AC susceptibility parameters for **1** at *T* = 2.0 K. ^a^

*B*_DC_/T	*R*(*χ*′)/%	*R*(*χ*″)/%	*χ*_S_	*χ*_LF_	*α*_LF_	*τ*_LF_/s	*χ*_HF_	*α*_HF_	*τ*_HF_/10^−6^ s	*x*_LF_
0.2	0.48	1.4	2.3(2)	4.8(3)	0.33(2)	0.08(1)	13.6(1)	0.07(1)	93(4)	0.22
0.4	0.49	2.4	0.63(18)	6.4(2)	0.38(1)	0.15(1)	12.7(1)	0.13(2)	107(5)	0.48
0.6	0.87	1.9	0.59(25)	7.4(3)	0.39(1)	0.25(1)	11.1(1)	0.11(3)	87(8)	0.64
0.8	0.79	2.4	0.02	7.1(8)	0.43(1)	0.44(2)	9.91(10)	0.14(6)	36(14)	0.72
1.0	0.93	2.5	0.08	6.2(17)	0.44(1)	0.59(3)	8.13(10)	0.20(13)	17.3	0.76

^a^ Obtained by a two-set Debye model; *χ* in units of 10^−6^ m^3^ mol^−1^; *x*_LF_—mole fraction of the low-frequency relaxing species.

**Table 4:** Temperature dependence of AC susceptibility components of **1** at *B*_DC_ = 0.6 T.^a^.

*T*/K	*R*(*χ*′)/%	*R*(*χ*″)/%	*χ*_S_	*χ*_LF_	*α*_LF_	*τ*_LF_/s	*χ*_HF_	*α*_HF_	*τ*_HF_/10^−6^ s	*x*_LF_
1.9	0.84	1.9	0.32(29)	7.1(3)	0.38(1)	0.25(1)	11.1(1)	0.14(3)	78(9)	0.63
2.3	0.92	2.9	0.44(34)	5.5(4)	0.37(1)	0.19(1)	9.9(1)	0.12(3)	76(9)	0.53
2.7	0.49	1.9	0.40(20)	3.9(2)	0.36(1)	0.16(1)	8.8(1)	0.13(1)	73(5)	0.41
3.1	0.49	2.2	0.41(20)	2.7(2)	0.34(1)	0.17(1)	8.0(1)	0.13(2)	71(4)	0.31
3.5	0.53	2.8	0.40(22)	1.9(2)	0.33(2)	0.22(2)	7.2(1)	0.10(2)	69(4)	0.22
3.9	0.52	3.7	0.50(24)	1.5(3)	0.34(4)	0.28(4)	6.6(1)	0.09(2)	66(4)	0.17
4.3	0.44	2.3	0.46(19)	1.1(2)	0.30(4)	0.30(4)	6.1(1)	0.08(1)	60(3)	0.11
4.7	0.41	1.8	0.61(18)	1.0(2)	0.33(6)	0.37(7)	5.6(1)	0.06(1)	55(3)	0.09

^a^ Obtained by a two-set Debye model; *χ* in units of 10^−6^ m^3^ mol^−1^.

## Data Availability

CCDC 2016605 (at 150 K) and 2016606 (at 298 K) for **1** contain the supplementary crystallographic data for this paper. These CIF data can also be obtained free of charge from the Cambridge Crystallographic Data Centre via http://www.ccdc.cam.ac.uk/data_request/cif.

## Supplementary Materials

The following are available online, Figure S1: Trinuclear cobalt compounds containing compartmental salen type Schiff base ligands [57,58,59]. Compounds are presented with their corresponding CCDC reference codes. Figure S2: Temperature dependence of the AC susceptibility components of **1** for frequencies *f* = 0.1–1500 Hz at *B*_DC_ = 0.6 T. Table S1: Crystallographic data of **1** at 150 and 298 K.

## References

[1] Benelli C., Gatteschi D. (2015). Introduction to Molecular Magnetism: From Transition Metals to Lanthanides.

[2] Linert W., Verdaguer M. (2003). Molecular Magnets Recent Highlights.

[3] Winpenny R.E.P. (2006). Single-Molecule Magnets and Related Phenomena.

[4] Rumberger E.M.W. (2004). Magnetization Dynamics in Single-Molecule Magnets. Ph.D. Thesis.

[5] Kahn O. (1993). Molecular Magnetism.

[6] Atanasov M., Aravena D., Suturina E., Bill E., Maganas D., Neese F. (2015). First principles approach to the electronic structure, magnetic anisotropy and spin relaxation in mononuclear 3d-transition metal single molecule magnets. Coord. Chem. Rev..

[7] Boča R. (1999). Theoretical Foundations of Molecular Magnetism in Current Methods in Inorganic Chemistry.

[8] Aharoni A. (2000). Introduction to the Theory of Ferromagnetism in International Series of Monographs on Physics.

[9] Kirchmayr H. (2001). Magnetic Anisotropy in Encyclopedia of Materials: Science and Technology.

[10] Vincent R., Klyatskaya S., Ruben M., Wernsdorfer W., Balestro F. (2012). Electronic read-out of a single nuclear spin using a molecular spin transistor. Nature.

[11] Wernsdorfer W., Sessoli R. (1999). Quantum phase interference and parity effects in magnetic molecular clusters. Science.

[12] Kahn O., Martinez C.J. (1998). Spin-transition polymers: From molecular materials toward memory devices. Science.

[13] Cavallini M., Gomez-Segura J., Ruiz-Molina D., Massi M., Albonetti C., Rovira C., Veciana J., Biscarini F. (2005). Magnetic information storage on polymers by using patterned single-molecule magnets. Angew. Chem. Int. Ed..

[14] Heber J. (2007). Information storage. Nature Mater..

[15] Tegus O., Brück E., Buschow K.H.J., de Boer F.R. (2001). Transition-metal-based magnetic refrigerants for room-temperature applications. Nature.

[16] Gschneidner K.A., Pecharsky V.K. (2008). Thirty years of near room temperature magnetic cooling: Where we are today and future prospects. Int. J. Refrig..

[17] Caneschi A., Gatteschi D., Sessoli R., Barra A.L., Brunel L.C., Guillot M. (1991). Alternating current susceptibility, high field magnetization, and millimeter band EPR evidence for a ground S = 10 state in [Mn_12_O_12_(CH_3_COO)_16_(H_2_O)_4_].2CH_3_COOH.4H_2_O. J. Am. Chem. Soc..

[18] Sessoli R., Tsai H.L., Schake A.R., Wang S., Vincent J.B., Folting K., Gatteschi D., Christou G., Hendrickson D.N. (1993). High-spin molecules: [Mn_12_O_12_(O_2_CR)_16_(H_2_O)_4_]. J. Am. Chem. Soc..

[19] Sessoli R., Gatteschi D., Caneschi A., Novak M.A. (1993). Magnetic bistability in a metal-ion cluster. Nature.

[20] Aubin S.M.J., Wemple M.W., Adams D.M., Tsai H.-L., Christou G., Hendrickson D.N. (1996). Distorted Mn^IV^Mn^III^_3_ cubane complexes as single-molecule magnets. J. Am. Chem. Soc..

[21] Kostakis G.E., Perlepes S.P., Blatov V.A., Proserpio D.M., Powell A.K. (2012). High-nuclearity cobalt coordination clusters: Synthetic, topological and magnetic aspects. Coord. Chem. Rev..

[22] Murrie M. (2010). Cobalt(II) single-molecule magnets. Chem. Soc. Rev..

[23] Stamatatos T.C., Christou G. (2009). Azide groups in higher oxidation state manganese cluster chemistry: From structural aesthetics to single-molecule magnets. Inorg. Chem..

[24] Mukherjee S., Abboud K.A., Wernsdorfer W., Christou G. (2013). Comproportionation reactions to Manganese(III/IV) pivalate clusters: A new half-integer spin single-molecule magnet. Inorg. Chem..

[25] Boča R., Rajnák C., Titiš J., Valigura D. (2017). Field supported slow magnetic relaxation in a mononuclear Cu(II) complex. Inorg. Chem..

[26] Cadiou C., Murrie M., Paulsen C., Villar V., Wernsdorfer W., Winpenny R.E.P. (2001). Studies of a nickel-based single molecule magnet: Resonant quantum tunnelling in an *S* = 12 molecule. Chem. Commun..

[27] Hazra S., Sasmal S., Fleck M., Grandjean F., Sougrati M.T., Ghosh M., Harris T.D., Long G.J., Mohanta S. (2011). Slow magnetic relaxation and electron delocalization in an *S* = ^9^/_2_ Iron(II/III) complex with two crystallographically inequivalent iron sites. J. Chem. Phys..

[28] Hoshino N., Ako A.M., Powell A.K., Oshio H. (2009). Molecular magnets containing wheel motifs. Inorg. Chem..

[29] Barra A.L., Caneschi A., Cornia A., de Biani F.F., Gatteschi D., Sangregorio C., Sessoli R., Sorace L. (1999). Single-molecule magnet behavior of a tetranuclear Iron(III) complex. The origin of slow magnetic relaxation in Iron(III) clusters. J. Am. Chem. Soc..

[30] Phonsri W., Harding P., Liu L., Telfer S.G., Murray K.S., Moubaraki B., Ross T.M., Jameson G.N.L., Harding D.J. (2017). Solvent modified spin crossover in an Iron(III) complex: Phase changes and an exceptionally wide hysteresis. Chem. Sci..

[31] Oshio H., Hoshino N., Ito T., Nakano M. (2004). Single-molecule magnets of ferrous cubes: Structurally controlled magnetic anisotropy. J. Am. Chem. Soc..

[32] McClain K.R., Gould C.A., Chakarawet K., Teat S.J., Groshens T.J., Long J.R., Harvey B.J. (2018). High-temperature magnetic blocking and magneto-structural correlations in a series of Dysprosium(III) metallocenium single-molecule magnets. Chem. Sci..

[33] Guo F.-S., Day B.M., Chen Y.-C., Tong M.-L., Mansikkamäki A., Layfield R.A. (2018). Magnetic hysteresis up to 80 kelvin in a dysprosium metallocene single-molecule magnet. Science.

[34] Goodwin C.A.P., Ortu F., Reta D., Chilton N.F., Mills D.P. (2017). Molecular magnetic hysteresis at 60 kelvin in dysprosocenium. Nature.

[35] Leuenberger M.N., Loss D. (2001). Quantum computing in molecular magnets. Nature.

[36] Lomjanský D., Moncoľ J., Rajnák C., Titiš J., Boča R. (2017). Field effects to slow magnetic relaxation in a mononuclear Ni(II) complex. Chem. Commun..

[37] Hazra S., Titiš J., Valigura D., Boca R., Mohanta S. (2016). Bis-phenoxido and bis-acetato bridged heteronuclear {Co^III^Dy^III^} single molecule magnets with two slow relaxation branches. Dalton Trans..

[38] Gregson M., Chilton N.F., Ariciu A.-M., Tuna F., Crowe I.F., Lewis W., Blake A.J., Collison D., McInnes E.J.L., Winpenny R.E.P. (2016). A monometallic lanthanide bis(methanediide) single molecule magnet with a large energy barrier and complex spin relaxation behaviour. Chem. Sci..

[39] Funes A.V., Carrella L., Rentschler E., Alborés P. (2014). {Co^III^_2_Dy^III^_2_} single molecule magnet with two resolved thermal activated magnetization relaxation pathways at zero field. Dalton Trans..

[40] Kaemmerer H., Baniodeh A., Peng Y., Moreno-Pineda E., Schulze M., Anson C.E., Wernsdorfer W., Schnack J., Powell A.K. (2020). Inorganic approach to stabilizing nanoscale toroidicity in a tetraicosanuclear Fe_18_Dy_6_ single molecule magnet. J. Am. Chem. Soc..

[41] Chandrasekhar V., Hossain S., Das S., Biswas S., Sutter J.-P. (2013). *Rhombus*-shaped tetranuclear [Ln_4_] complexes [Ln = Dy(III) and Ho(III)]: Synthesis, structure, and SMM behavior. Inorg. Chem..

[42] Boča R., Miklovic J., Titiš J. (2014). Simple mononuclear Cobalt(II) complex: A single-molecule magnet showing two slow relaxation processes. Inorg. Chem..

[43] Mitsuhashi R., Pedersen K.S., Ueda T., Suzuki T., Bendix J., Mikuriya M. (2018). Field-induced single-molecule magnet behavior in ideal trigonal antiprismatic Cobalt(II) complexes: Precise geometrical control by a hydrogen-bonded rigid metalloligand. Chem. Commun..

[44] Zadrozny J.M., Long J.R. (2011). Slow magnetic relaxation at zero field in the tetrahedral complex [Co(SPh)_4_]^2–^. J. Am. Chem. Soc..

[45] Buvaylo E.A., Kokozay V.N., Vassilyeva Y.O., Skelton B.W., Ozarowski A., Titiš J., Vranovičová B., Boča R. (2017). Field-assisted slow magnetic relaxation in a six-coordinate Co(II)–Co(III) complex with large negative anisotropy. Inorg. Chem..

[46] Ferguson A., Parkin A., Sanchez-Benitez J., Kamenev K., Wernsdorfer W., Murrie M. (2007). A mixed-valence Co_7_ single-molecule magnet with *C*_3_ symmetry. Chem. Commun..

[47] Rajnák C., Varga F., Titiš J., Moncoľ J., Boča R. (2018). Octahedral–tetrahedral systems [Co(*dppm^O^*^,*O*^)_3_]^2+^[CoX_4_]^2–^ showing slow magnetic relaxation with two relaxation modes. Inorg. Chem..

[48] Varga F., Rajnák C., Titiš J., Moncoľ J., Boča R. (2017). Slow magnetic relaxation in a Co(II) octahedral–tetrahedral system formed of a [CoL_3_]^2+^ core with L = bis(diphenylphosphanoxido) methane and tetrahedral [CoBr_4_]^2−^ counter anions. Dalton Trans..

[49] Valigura D., Rajnák C., Moncoľ J., Titiš J., Boča R. (2017). A mononuclear Co(II) complex formed from pyridinedimethanol with manifold slow relaxation channels. Dalton Trans..

[50] Rajnák C., Dlháň Ľ., Moncol J., Titiš J., Boča R. (2018). Slow magnetic relaxation in a μ_1,1′_-azido cobalt(II) methylquinoline chain complex. Dalton Trans..

[51] Zhu Y.-Y., Cui C., Zhang Y.-Q., Jia J.-H., Guo X., Gao C., Qian K., Jiang S.-D., Wang B.-W., Wang Z.-M. (2013). Zero-field slow magnetic relaxation from single Co(II) ion: A transition metal single-molecule magnet with high anisotropy barrier. Chem. Sci..

[52] Hazra S., Mohanta S. (2019). Metal–tin derivatives of compartmental Schiff Bases: Synthesis, structure and application. Coord. Chem. Rev..

[53] Hazra S., Chakraborty P., Mohanta S. (2016). Heterometallic Copper(II)–Tin(II/IV) salts, cocrystals, and salt cocrystals: Selectivity and structural diversity depending on ligand substitution and the metal oxidation state. Cryst. Growth Des..

[54] Hazra S., Meyrelles R., Charmier A.J., Rijo P., Guedes da Silva M.F.C., Pombeiro A.J.L. (2016). N–H⋯O and N–H⋯Cl supported 1D chains of heterobimetallic Cu^II^/Ni^II^–Sn^IV^ cocrystals. Dalton Trans..

[55] Hazra S., Martins N.M.R., Kuznetsov M., Guedes da Silva M.F.C., Pombeiro A.J.L. (2017). Flexibility and lability of a phenyl ligand in hetero-organometallic 3d metal–Sn(IV) compounds and their catalytic activity in Baeyer–Villiger oxidation of cyclohexanone. Dalton Trans..

[56] Pfeiffer P., Breith E., Lübbe E., Tsumaki T. (1933). Tricyclische orthokondensierte Nebenvalenzringe. Justus Liebigs Ann. Chem..

[57] Tang J., Huang F., Wei Y., Bian H., Zhang W., Liang H. (2016). Bovine serum albumin–Cobalt(II) Schiff base complex hybrid: An efficient artificial metalloenzyme for enantioselective sulfoxidation using hydrogen peroxide. Dalton Trans..

[58] Mechi L., Siega P., Dreos R., Zangrando E., Randaccio L. (2009). Crystal structures and solution behavior of paramagnetic, trinuclear, mixed-valent cobalt complexes with Salen-type ligands. Eur. J. Inorg. Chem..

[59] Groom C.R., Bruno I.J., Lightfoot M.P., Ward S.C. (2016). The Cambridge structural database. Acta Cryst..

[60] Jarzembska K.N., Kamiński R., Durka K., Kubsik M. (2017). Engineering of solvatomorphs of the luminescent complex of *ortho*-phenylenediboronic acid and 8-hydroxyquinoline. Cryst. Growth Des..

[61] Hazra S., Guedes da Silva M.F.C., Karmakar A., Pombeiro A.J.L. (2015). 1D hacksaw chain bipyridine–sulfonate Schiff base-dicopper(II) as a host for variable solvent guests. RSC Adv..

[62] Chandrasekhar V., Dey A., Mota A.J., Colacio E. (2013). Slow magnetic relaxation in Co(III)–Co(II) mixed-valence dinuclear complexes with a Co^II^O_5_X (X = Cl, Br, NO_3_) distorted-octahedral coordination sphere. Inorg. Chem..

[63] Shiga T., Onuki T., Matsumoto T., Nojiri H., Newton G.N., Hoshino N., Oshio H. (2009). Undecanuclear mixed-valence 3d–4f bimetallic clusters. Chem. Commun..

[64] Brechin E.K., Huffman J.C., Christou G., Yoo J., Nakano M., Hendrickson D.N. (1999). A new class of single-molecule magnets: Mixed-valent [Mn_4_(O_2_CMe)_2_(Hpdm)_6_][ClO_4_]_2_ with an *S* = 8 ground state. Chem. Commun..

[65] Zhang Z.-M., Yao S., Li Y.-G., Wu H.-H., Wang Y.-H., Rouzières M., Clérac R., Su Z.-M., Wang E.-B. (2013). A polyoxometalate-based single-molecule magnet with a mixed-valent {Mn^IV^_2_Mn^III^_6_Mn^II^_4_} core. Chem. Commun..

[66] Yoo J., Yamaguchi A., Nakano M., Krzystek J., Streib W.E., Brunel L.-C., Ishimoto H., Christou G., Hendrickson D.N. (2001). Mixed-valence tetranuclear manganese single-molecule magnets. Inorg. Chem..

[67] Stamatatos T.C., Nastopoulos V., Tasiopoulos A.J., Moushi E.E., Wernsdorfer W., Christou G., Perlepes S.P. (2008). High nuclearity single-molecule magnets: A mixed-valence Mn_26_ cluster containing the Di-2-pyridylketone diolate dianion. Inorg. Chem..

[68] Ako A.M., Mereacre V., Hewitt I.J., Clérac R., Lecren L., Anson C.E., Powell A.K. (2006). Enhancing single molecule magnet parameters. Synthesis, crystal structures and magnetic properties of mixed-valent Mn_4_ SMMs. J. Mater. Chem..

[69] Boča R. (2012). A Handbook of Magnetochemical Formulae.

[70] (2012). APEX2, SMART, SAINT & SADABS: Bruker.

[71] Sheldrick G.M. (2015). Crystal structure refinement with SHELXL. Acta Cryst..

[72] Farrugia L.J. (2012). WinGX and ORTEP for Windows: An update. J. Appl. Cryst..

[73] Spek A.L. (2015). PLATON SQUEEZE: A tool for the calculation of the disordered solvent contribution to the calculated structure factors. Acta Cryst..

[74] Neese F. (2012). The ORCA program system, WIREs. Comput. Mol. Sci..

[75] Neese F. (2017). ORCA—An Ab Initio, Density Functional and Semi-empirical Program Package, Version 4.0.0.

[76] Atanasov M., Ganyushin D., Pantazis D.A., Sivalingam K., Neese F. (2011). Detailed ab initio first-principles study of the magnetic anisotropy in a family of trigonal pyramidal Iron(II) pyrrolide complexes. Inorg. Chem..

[77] Angeli C., Borini S., Cestari M., Cimiraglia R. (2004). A quasidegenerate formulation of the second order n-electron valence state perturbation theory approach. J. Chem. Phys..

[78] Neese F. (2005). Efficient and accurate approximations to the molecular spin-orbit coupling operator and their use in molecular *g*-tensor calculations. J. Chem. Phys..

[79] Ganyushin D., Neese F. (2006). First-principles calculations of zero-field splitting parameters. J. Chem. Phys..

[80] Neese F. (2007). Calculation of the zero-field splitting tensor on the basis of hybrid density functional and Hartree-Fock theory. J. Chem. Phys..

