# Supplementary material for: A Mixed Valence CoIICoIII2 Field-Supported Single Molecule Magnet: Solvent-Dependent Structural Variation
Source: Molecules. 2021 Feb 18;26(4):1060. doi: 10.3390/molecules26041060 (PMC7922140; doi:10.3390/molecules26041060)

checkCIF/PLATON report

Structure factors have been supplied for datablock(s) SH960LT

THIS REPORT IS FOR GUIDANCE ONLY. IF USED AS PART OF A REVIEW PROCEDURE FOR PUBLICATION, IT SHOULD NOT REPLACE THE EXPERTISE OF AN EXPERIENCED CRYSTALLOGRAPHIC REFEREE.

No syntax errors found.   CIF dictionary   Interpreting this report

Datablock: SH960LT

```
Bond precision:  C-C = 0.0038 A          Wavelength=0.71073

Cell:             a=13.4175(13)      b=23.569(2)     c=27.469(3)
                  alpha=90           beta=90         gamma=90
Temperature:      150 K
```

	Calculated	Reported
Volume	8686.7(15)	8686.8(15)
Space group	P b c a	P b c a
Hall group	-P 2ac 2ab	-P 2ac 2ab
Moiety formula	C44 H38 Cl2 Co3 N4 O4, C3 H6 O, H2 O	?
Sum formula	C47 H46 Cl2 Co3 N4 O6	C47 H46 Cl2 Co3 N4 O6
Mr	1010.57	1010.57
Dx,g cm-3	1.545	1.545
Z	8	8
Mu (mm-1)	1.310	1.310
F000	4152.0	4152.0
F000’	4164.02	
h,k,lmax	17,30,35	17,30,35
Nref	9676	9648
Tmin,Tmax	0.754,0.822	0.747,0.819
Tmin’	0.750	

```
Correction method= # Reported T Limits: Tmin=0.747 Tmax=0.819
AbsCorr = MULTI-SCAN

Data completeness= 0.997          Theta(max)= 27.192

R(reflections)= 0.0334( 7976)     wR2(reflections)= 0.0894( 9648)

S = 1.096                 Npar= 565
```

The following ALERTS were generated. Each ALERT has the format
test-name_ALERT_alert-type_alert-level.
Click on the hyperlinks for more details of the test.

Alert level B

```
PLAT420_ALERT_2_B D-H Without Acceptor       O6       --H6A      .     Please Check
```

Alert level C

```
PLAT220_ALERT_2_C NonSolvent Resd 1  C   Ueq(max) / Ueq(min) Range        3.2 Ratio
PLAT260_ALERT_2_C Large Average Ueq of Residue Including       O6      0.103 Check
PLAT905_ALERT_3_C Negative K value in the Analysis of Variance ...    -0.162 Report
PLAT910_ALERT_3_C Missing # of FCF Reflection(s) Below Theta(Min).         5 Note
PLAT976_ALERT_2_C Check Calcd Resid. Dens.  0.59A   From O6            -0.54 eA-3
PLAT976_ALERT_2_C Check Calcd Resid. Dens.  0.73A   From O6            -0.52 eA-3
PLAT976_ALERT_2_C Check Calcd Resid. Dens.  0.63A   From O6            -0.43 eA-3
PLAT976_ALERT_2_C Check Calcd Resid. Dens.  0.74A   From O6            -0.42 eA-3
PLAT977_ALERT_2_C Check Negative Difference Density on H47B            -0.36 eA-3
PLAT977_ALERT_2_C Check Negative Difference Density on H47C            -0.49 eA-3
```

Alert level G

```
PLAT002_ALERT_2_G Number of Distance or Angle Restraints on AtSite         3 Note
PLAT066_ALERT_1_G Predicted and Reported Tmin&Tmax Range Identical         ? Check
PLAT083_ALERT_2_G SHELXL Second Parameter in WGHT  Unusually Large     13.63 Why ?
PLAT172_ALERT_4_G The CIF-Embedded .res File Contains DFIX Records         2 Report
PLAT173_ALERT_4_G The CIF-Embedded .res File Contains DANG Records         1 Report
PLAT232_ALERT_2_G Hirshfeld Test Diff (M-X)  Co2      --Cl2      .       6.3 s.u.
PLAT232_ALERT_2_G Hirshfeld Test Diff (M-X)  Co3      --Cl1      .      10.0 s.u.
PLAT232_ALERT_2_G Hirshfeld Test Diff (M-X)  Co3      --Cl2      .       8.3 s.u.
PLAT380_ALERT_4_G Incorrectly? Oriented X(sp2)-Methyl Moiety .....       C46 Check
PLAT380_ALERT_4_G Incorrectly? Oriented X(sp2)-Methyl Moiety .....       C47 Check
PLAT794_ALERT_5_G Tentative Bond Valency for Co1       (III)     .      3.10 Info
PLAT794_ALERT_5_G Tentative Bond Valency for Co2       (III)     .      3.14 Info
PLAT794_ALERT_5_G Tentative Bond Valency for Co3       (II)      .      2.01 Info
PLAT860_ALERT_3_G Number of Least-Squares Restraints .............         3 Note
PLAT883_ALERT_1_G No Info/Value for _atom_sites_solution_primary .     Please Do !
PLAT912_ALERT_4_G Missing # of FCF Reflections Above STh/L=  0.600        23 Note
PLAT913_ALERT_3_G Missing # of Very Strong Reflections in FCF ....         2 Note
PLAT965_ALERT_2_G The SHELXL WEIGHT Optimisation has not Converged     Please Check
PLAT978_ALERT_2_G Number C-C Bonds with Positive Residual Density.        14 Info
```

---

0 **ALERT level A** = Most likely a serious problem - resolve or explain
1 **ALERT level B** = A potentially serious problem, consider carefully
10 **ALERT level C** = Check. Ensure it is not caused by an omission or oversight
19 **ALERT level G** = General information/check it is not something unexpected

2 ALERT type 1 CIF construction/syntax error, inconsistent or missing data
16 ALERT type 2 Indicator that the structure model may be wrong or deficient
4 ALERT type 3 Indicator that the structure quality may be low
5 ALERT type 4 Improvement, methodology, query or suggestion
3 ALERT type 5 Informative message, check

It is advisable to attempt to resolve as many as possible of the alerts in all categories. Often the minor alerts point to easily fixed oversights, errors and omissions in your CIF or refinement strategy, so attention to these fine details can be worthwhile. In order to resolve some of the more serious problems it may be necessary to carry out additional measurements or structure refinements. However, the purpose of your study may justify the reported deviations and the more serious of these should normally be commented upon in the discussion or experimental section of a paper or in the "special_details" fields of the CIF. checkCIF was carefully designed to identify outliers and unusual parameters, but every test has its limitations and alerts that are not important in a particular case may appear. Conversely, the absence of alerts does not guarantee there are no aspects of the results needing attention. It is up to the individual to critically assess their own results and, if necessary, seek expert advice.

Publication of your CIF in IUCr journals

A basic structural check has been run on your CIF. These basic checks will be run on all CIFs submitted for publication in IUCr journals (*Acta Crystallographica*, *Journal of Applied Crystallography*, *Journal of Synchrotron Radiation*); however, if you intend to submit to *Acta Crystallographica Section C* or *E* or *IUCrData*, you should make sure that full publication checks are run on the final version of your CIF prior to submission.

Publication of your CIF in other journals

Please refer to the *Notes for Authors* of the relevant journal for any special instructions relating to CIF submission.

PLATON version of 04/06/2020; check.def file version of 02/06/2020

Datablock SH960LT - ellipsoid plot

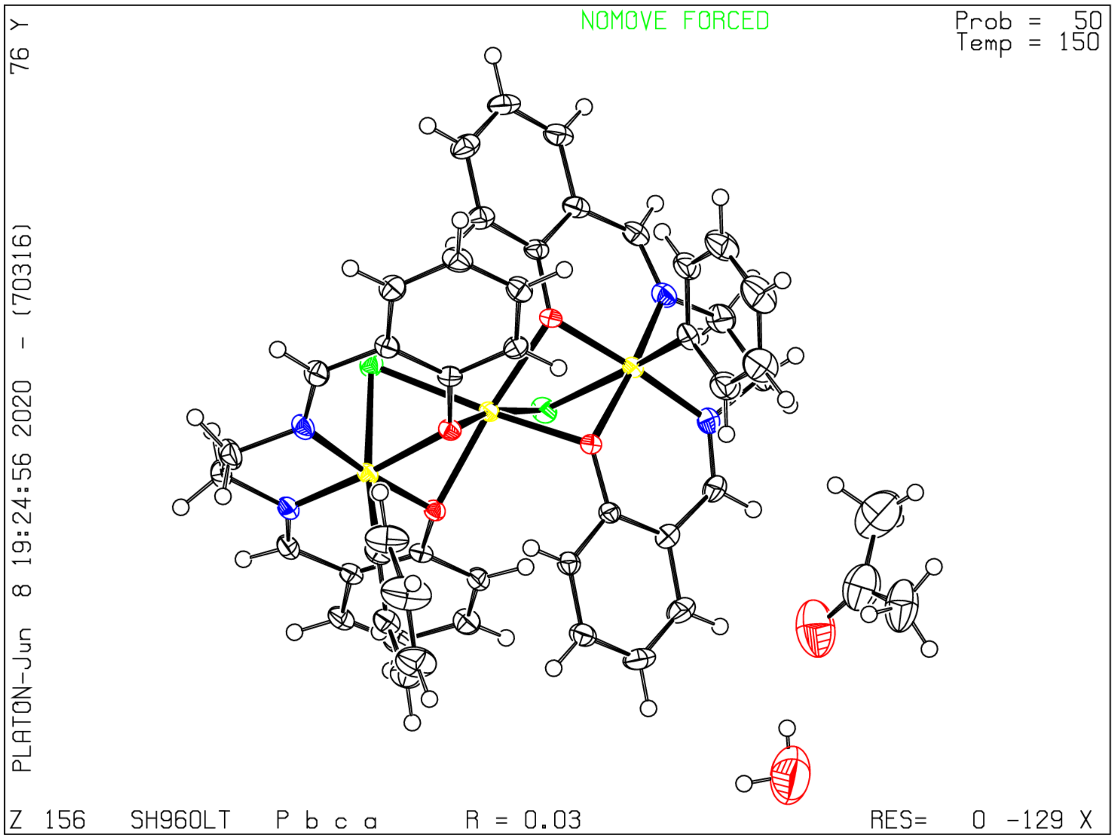

Supplement: Supplementary file 1 [file molecules-26-01060-s001.zip › molecules-1112883-supplementary/checkcif-Compound1-LT.pdf]
