# Supplementary material for: A Mixed Valence CoIICoIII2 Field-Supported Single Molecule Magnet: Solvent-Dependent Structural Variation
Source: Molecules. 2021 Feb 18;26(4):1060. doi: 10.3390/molecules26041060 (PMC7922140; doi:10.3390/molecules26041060)

checkCIF/PLATON report

Structure factors have been supplied for datablock(s) SH960RT

THIS REPORT IS FOR GUIDANCE ONLY. IF USED AS PART OF A REVIEW PROCEDURE FOR PUBLICATION, IT SHOULD NOT REPLACE THE EXPERTISE OF AN EXPERIENCED CRYSTALLOGRAPHIC REFEREE.

No syntax errors found. CIF dictionary Interpreting this report

Datablock: SH960RT

```
Bond precision:  C-C = 0.0070 A          Wavelength=0.71073

Cell:            a=13.4805(11)     b=23.6077(19)     c=27.937(2)
                 alpha=90          beta=90           gamma=90
Temperature:     298 K
```

	Calculated	Reported
Volume	8890.8(12)	8890.9(13)
Space group	P b c a	P b c a
Hall group	-P 2ac 2ab	-P 2ac 2ab
Moiety formula	C44 H38 Cl2 Co3 N4 O4 [+ solvent]	?
Sum formula	C44 H38 Cl2 Co3 N4 O4 [+ solvent]	C47 H46 Cl2 Co3 N4 O6
Mr	934.47	1010.57
Dx,g cm-3	1.396	1.510
Z	8	8
Mu (mm-1)	1.271	1.280
F000	3816.0	4152.0
F000’	3827.80	
h,k,lmax	16,28,34	16,28,34
Nref	8489	8440
Tmin,Tmax	0.758,0.825	0.749,0.820
Tmin’	0.755	

```
Correction method= # Reported T Limits: Tmin=0.749 Tmax=0.820
AbsCorr = MULTI-SCAN

Data completeness= 0.994          Theta(max)= 25.731

R(reflections)= 0.0559( 6608)     wR2(reflections)= 0.1147( 8440)

S = 1.124                  Npar= 514
```

The following ALERTS were generated. Each ALERT has the format
test-name_ALERT_alert-type_alert-level.
Click on the hyperlinks for more details of the test.

Alert level B

```
PLAT990_ALERT_1_B Deprecated .res/.hkl Input Style SQUEEZE Job ...          ! Note
```

Alert level C

```
PLAT220_ALERT_2_C NonSolvent Resd 1  C   Ueq(max) / Ueq(min) Range        4.0 Ratio
PLAT241_ALERT_2_C High   'MainMol' Ueq as Compared to Neighbors of        C41 Check
PLAT341_ALERT_3_C Low Bond Precision on  C-C Bonds ...............    0.00705 Ang.
PLAT906_ALERT_3_C Large K Value in the Analysis of Variance ......     13.922 Check
PLAT906_ALERT_3_C Large K Value in the Analysis of Variance ......      2.875 Check
PLAT910_ALERT_3_C Missing # of FCF Reflection(s) Below Theta(Min).          5 Note
PLAT911_ALERT_3_C Missing FCF Refl Between Thmin & STh/L=    0.600         20 Report
```

Alert level G

```
FORMU01_ALERT_2_G  There is a discrepancy between the atom counts in the
            _chemical_formula_sum and the formula from the _atom_site* data.
            Atom count from _chemical_formula_sum:C47 H46 Cl2 Co3 N4 O6
            Atom count from the _atom_site data:  C44 H38 Cl2 Co3 N4 O4
CELLZ01_ALERT_1_G Difference between formula and atom_site contents detected.
CELLZ01_ALERT_1_G ALERT: Large difference may be due to a
            symmetry error - see SYMMG tests
           From the CIF: _cell_formula_units_Z    8
           From the CIF: _chemical_formula_sum  C47 H46 Cl2 Co3 N4 O6
           TEST: Compare cell contents of formula and atom_site data

           atom    Z*formula  cif sites diff
           C        376.00    352.00   24.00
           H        368.00    304.00   64.00
           Cl        16.00     16.00    0.00
           Co        24.00     24.00    0.00
           N         32.00     32.00    0.00
           O         48.00     32.00   16.00
PLAT041_ALERT_1_G Calc. and Reported SumFormula    Strings  Differ     Please Check
PLAT066_ALERT_1_G Predicted and Reported Tmin&Tmax Range Identical          ? Check
PLAT068_ALERT_1_G Reported F000 Differs from Calcd (or Missing)...     Please Check
PLAT083_ALERT_2_G SHELXL Second Parameter in WGHT  Unusually Large      20.91 Why ?
PLAT232_ALERT_2_G Hirshfeld Test Diff (M-X)  Co2      --Cl2      .        5.5 s.u.
PLAT232_ALERT_2_G Hirshfeld Test Diff (M-X)  Co3      --Cl1      .        5.3 s.u.
PLAT606_ALERT_4_G VERY LARGE Solvent Accessible VOID(S) in Structure        ! Info
PLAT794_ALERT_5_G Tentative Bond Valency for Co1       (III)     .       3.10 Info
PLAT794_ALERT_5_G Tentative Bond Valency for Co2       (III)     .       3.17 Info
PLAT794_ALERT_5_G Tentative Bond Valency for Co3       (II)      .       2.00 Info
PLAT869_ALERT_4_G ALERTS Related to the Use of SQUEEZE Suppressed           ! Info
PLAT883_ALERT_1_G No Info/Value for _atom_sites_solution_primary .     Please Do !
PLAT912_ALERT_4_G Missing # of FCF Reflections Above STh/L=  0.600         24 Note
PLAT913_ALERT_3_G Missing # of Very Strong Reflections in FCF ....          3 Note
PLAT933_ALERT_2_G Number of OMIT Records in Embedded .res File ...          1 Note
PLAT961_ALERT_5_G Dataset Contains no Negative Intensities .......     Please Check
PLAT965_ALERT_2_G The SHELXL WEIGHT Optimisation has not Converged     Please Check
PLAT978_ALERT_2_G Number C-C Bonds with Positive Residual Density.          2 Info
```

---

0 **ALERT level A** = Most likely a serious problem - resolve or explain
1 **ALERT level B** = A potentially serious problem, consider carefully
7 **ALERT level C** = Check. Ensure it is not caused by an omission or oversight

```
21 ALERT level G = General information/check it is not something unexpected

 7 ALERT type 1 CIF construction/syntax error, inconsistent or missing data
 9 ALERT type 2 Indicator that the structure model may be wrong or deficient
 6 ALERT type 3 Indicator that the structure quality may be low
 3 ALERT type 4 Improvement, methodology, query or suggestion
 4 ALERT type 5 Informative message, check
```

It is advisable to attempt to resolve as many as possible of the alerts in all categories. Often the minor alerts point to easily fixed oversights, errors and omissions in your CIF or refinement strategy, so attention to these fine details can be worthwhile. In order to resolve some of the more serious problems it may be necessary to carry out additional measurements or structure refinements. However, the purpose of your study may justify the reported deviations and the more serious of these should normally be commented upon in the discussion or experimental section of a paper or in the "special_details" fields of the CIF. checkCIF was carefully designed to identify outliers and unusual parameters, but every test has its limitations and alerts that are not important in a particular case may appear. Conversely, the absence of alerts does not guarantee there are no aspects of the results needing attention. It is up to the individual to critically assess their own results and, if necessary, seek expert advice.

Publication of your CIF in IUCr journals

A basic structural check has been run on your CIF. These basic checks will be run on all CIFs submitted for publication in IUCr journals (*Acta Crystallographica*, *Journal of Applied Crystallography*, *Journal of Synchrotron Radiation*); however, if you intend to submit to *Acta Crystallographica Section C* or *E* or *IUCrData*, you should make sure that full publication checks are run on the final version of your CIF prior to submission.

Publication of your CIF in other journals

Please refer to the *Notes for Authors* of the relevant journal for any special instructions relating to CIF submission.

PLATON version of 04/06/2020; check.def file version of 02/06/2020

Datablock SH960RT - ellipsoid plot

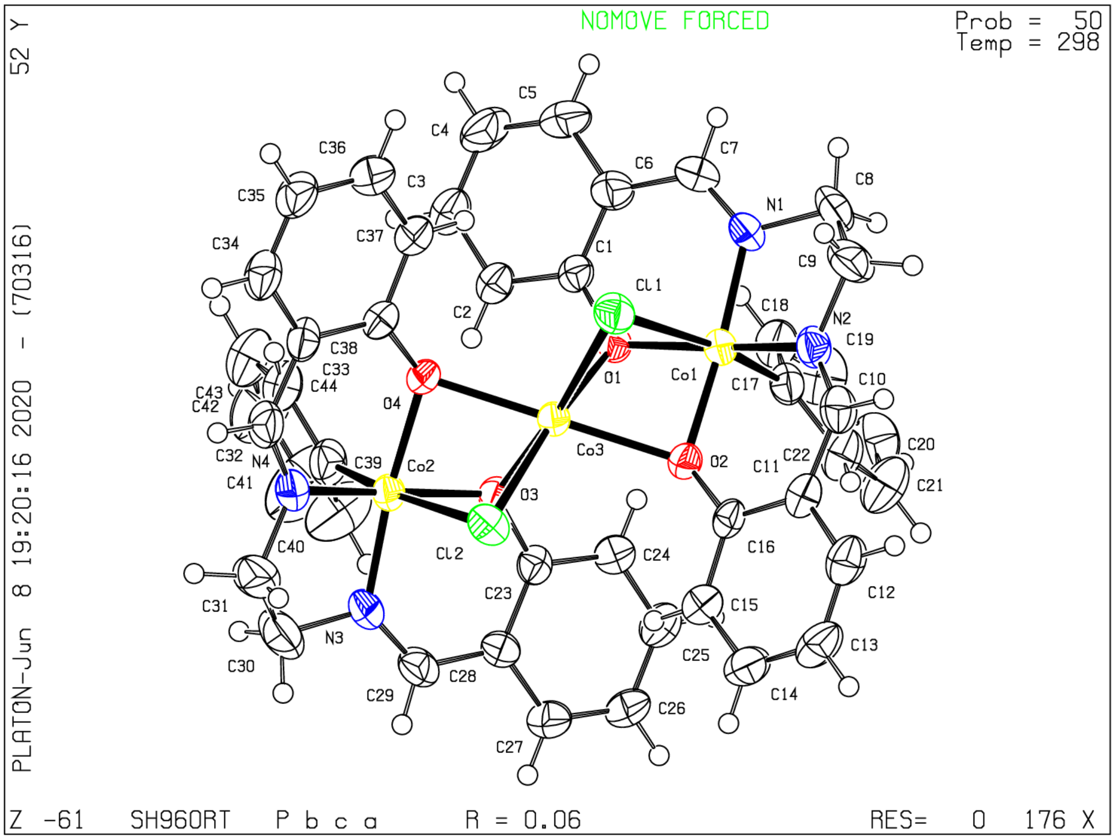

Supplement: Supplementary file 1 [file molecules-26-01060-s001.zip › molecules-1112883-supplementary/checkcif-Compound1-RT.pdf]
