# Supplementary material for: A Mixed Valence CoIICoIII2 Field-Supported Single Molecule Magnet: Solvent-Dependent Structural Variation
Source: Molecules. 2021 Feb 18;26(4):1060. doi: 10.3390/molecules26041060 (PMC7922140; doi:10.3390/molecules26041060)
Supplement: Supplementary file 1 [file molecules-26-01060-s001.zip › molecules-1112883-supplementary/Supplementary Materials.pdf]

Supplementary Materials

for

A Mixed Valence $Co^{II}Co^{III}_2$ Field Supported Single Molecule Magnet: Solvent Dependent Structural Variation

Susanta Hazra [1,*], C. Rajnák [2], Ján Titiš [2], M. Fátima C. Guedes da Silva [1], Roman Boča [2] and Armando J. L. Pombeiro [1,3,*]

[1] Centro de Química Estrutural, Instituto Superior Técnico, Universidade de Lisboa, Av. Rovisco Pais, 1049–001, Lisbon, Portugal.
[2] Department of Chemistry, Faculty of Natural Sciences, University of SS Cyril and Methodius, 91701 Trnava, Slovakia.
[3] Peoples' Friendship University of Russia (RUDN University), 6 Miklukho-Maklaya Street, Moscow, 117198, Russian Federation.
* Correspondence: h.susanta@gmail.com (S.H.); pombeiro@tecnico.ulisboa.pt (A.J.L.P.).

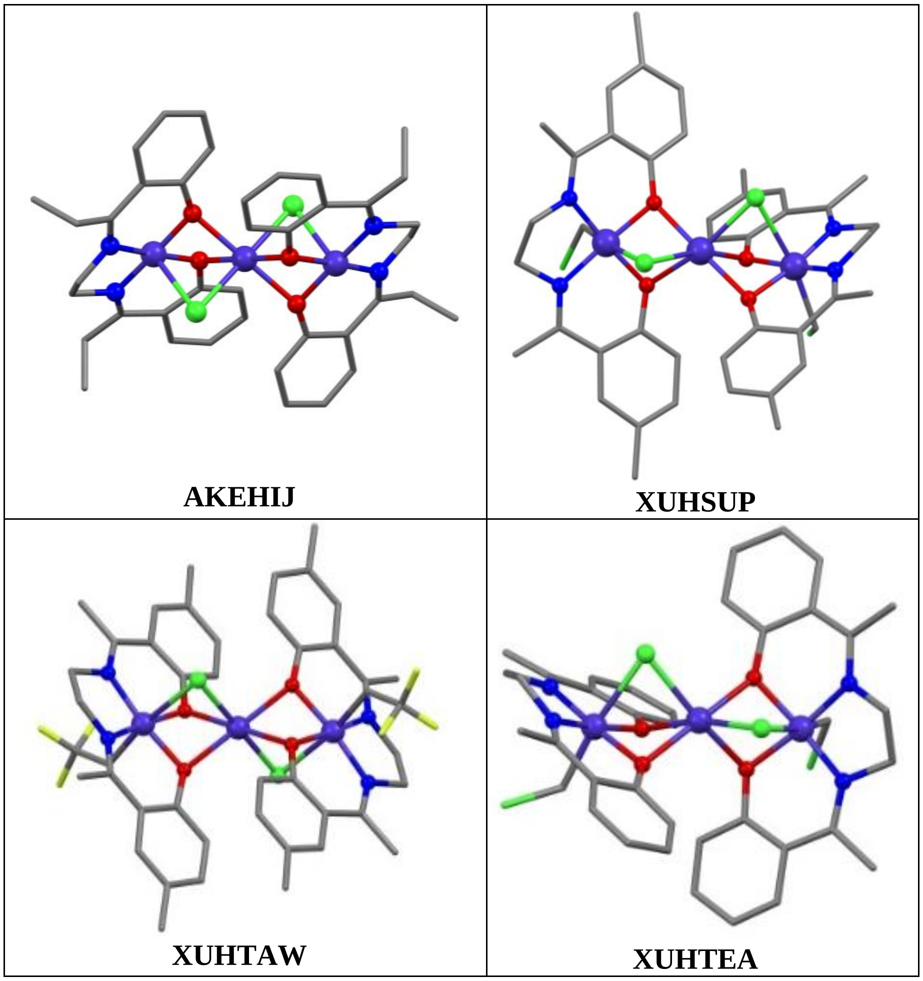

Figure S1. Trinuclear cobalt compounds containing compartmental salen type Schiff base ligands [57-59]. Compounds are presented with their corresponding CCDC reference codes.

The real and imaginary components of the AC susceptibility in terms of the extended two-set Debye model

$$\chi'(\omega) = \chi_S + (\chi_{T1} - \chi_S)\frac{1 + (\omega\tau_1)^{1-\alpha_1}\sin(\pi\alpha_1/2)}{1 + 2(\omega\tau_1)^{1-\alpha_1}\sin(\pi\alpha_1/2) + (\omega\tau_1)^{2-2\alpha_1}}$$
$$+(\chi_{T2} - \chi_{T1})\frac{1 + (\omega\tau_2)^{1-\alpha_2}\sin(\pi\alpha_2/2)}{1 + 2(\omega\tau_2)^{1-\alpha_2}\sin(\pi\alpha_2/2) + (\omega\tau_2)^{2-2\alpha_2}} + \ldots$$

$$\chi''(\omega) = (\chi_{T1} - \chi_S)\frac{(\omega\tau_1)^{1-\alpha_1}\cos(\pi\alpha_1/2)}{1 + 2(\omega\tau_1)^{1-\alpha_1}\sin(\pi\alpha_1/2) + (\omega\tau_1)^{2-2\alpha_1}}$$
$$+(\chi_{T2} - \chi_{T1})\frac{(\omega\tau_2)^{1-\alpha_2}\cos(\pi\alpha_2/2)}{1 + 2(\omega\tau_2)^{1-\alpha_2}\sin(\pi\alpha_2/2) + (\omega\tau_2)^{2-2\alpha_2}} + \ldots$$

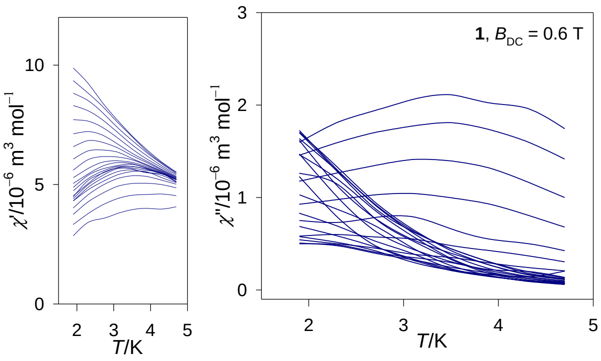

Figure S2. Temperature dependence of the AC susceptibility components of **1** for frequencies *f* = 0.1 – 1500 Hz at B_{DC} = 0.6 T.

Table S1. Crystallographic data of **1** at 150 and 298 K.

T/K	150(2)	298(2)
Formula	$C_{47}H_{46}Cl_2Co_3N_4O_6$	$C_{47}H_{46}Cl_2Co_3N_4O_6$
FW	1010.57	1010.57
Crystal system	Orthorhombic	Orthorhombic
Space group	*P*bca	*P*bca
a/Å	13.4175(13)	13.4805(11)
b/Å	23.569(2)	23.6077(19)
c/Å	27.469(3)	27.937(2)
α / β / γ /°	90.00	90.00
V/Å^3	8686.8(15)	8890.9(13)
Z	8	8
θ/°	2.277 – 27.192	2.259 – 25.731
μ (Mo Kα)/mm^{-1}	1.310	1.280
ρ_{calcd}/g cm^{-3}	1.545	1.510
F(000)	4152	4152
Index ranges	$-16<h<17$	$-16<h<16$
	$-30<k<30$	$-28<k<25$
	$-35<l<35$	$-34<l<34$
Rfs. collected	160792	103125
Rfs. unique/observed	9648 / 7976	8440 / 6608
R_{int}	0.0561	0.0948
R_1[a]/ wR_2[b] [I > 2σ(I)]	0.0334 / 0.0789	0.0559 / 0.1076
R_1[a]/ wR_2[b] [for all F_o^2]	0.0480 / 0.0894	0.0781 / 0.1147
GOF on F^2	1.096	1.124

.
